# *Karnatukul* (Serpent’s Glen): A new chronology for the oldest site in Australia’s Western Desert

**DOI:** 10.1371/journal.pone.0202511

**Published:** 2018-09-19

**Authors:** Jo McDonald, Wendy Reynen, Fiona Petchey, Kane Ditchfield, Chae Byrne, Dorcas Vannieuwenhuyse, Matthias Leopold, Peter Veth

**Affiliations:** 1 Centre for Rock Art Research + Management, Faculty of Arts, Business, Law and Education, University of Western Australia, Crawley, WA, Australia; 2 Archaeology Department, Faculty of Arts, Business, Law and Education, University of Western Australia, Crawley, WA, Australia; 3 Waikato Dating Laboratory, University of Waikato, Hamilton, New Zealand; 4 School of Agriculture and Environment, University of Western Australia, Crawley, WA, Australia; Max Planck Institute for the Science of Human History, GERMANY

## Abstract

The re-excavation of *Karnatukul* (Serpent’s Glen) has provided evidence for the human occupation of the Australian Western Desert to before 47,830 cal. BP (modelled median age). This new sequence is 20,000 years older than the previous known age for occupation at this site. Re-excavation of *Karnatukul* aimed to contextualise the site’s painted art assemblage. We report on analyses of assemblages of stone artefacts and pigment art, pigment fragments, anthracology, new radiocarbon dates and detailed sediment analyses. Combined these add significantly to our understanding of this earliest occupation of Australia’s Western Desert. The large lithic assemblage of over 25,000 artefacts includes a symmetrical geometric backed artefact dated to 45,570–41,650 cal. BP. The assemblage includes other evidence for hafting technology in its earliest phase of occupation. This research recalibrates the earliest Pleistocene occupation of Australia’s desert core and confirms that people remained in this part of the arid zone during the Last Glacial Maximum. Changes in occupation intensity are demonstrated throughout the sequence: at the late Pleistocene/Holocene transition, the mid-Holocene and then during the last millennium. *Karnatukul* documents intensive site use with a range of occupation activities and different signalling behaviours during the last 1,000 years. This correlation of rock art and occupation evidence refines our understanding of how Western Desert peoples have inscribed their landscapes in the recent past, while the newly described occupation sequence highlights the dynamic adaptive culture of the first Australians, supporting arguments for their rapid very early migration from the coasts and northern tropics throughout the arid interior of the continent.

## Introduction

Serpent’s Glen rockshelter, known as *Karnatukul* to its traditional custodians, is in the Carnarvon Ranges (more properly, *Katjarra)*. Serpent’s Glen rockshelter was the first Pleistocene site identified in the Western Desert with a (calibrated) 26,000 year old basal date [1]. Initially identified as having a two-phase occupation sequence, this site was central to developing arguments about arid zone demographic restructuring during the Last Glacial Maximum (‘LGM’), and provided a glimpse into the earliest occupation of the western Desert, in a landscape where most occupation evidence dates to the Holocene [2–5]. The site’s Holocene occupation sequence was previously dated to the last 5,000 years with the most intensive phase indistinguishable-from-modern [1]. The original excavated assemblage was lost during the January 2003 ACT bushfires, when ANU’s data repository (along with 500 homes) was destroyed (Sue O’Connor, pers. comm., 2006).

**Fig 1:**
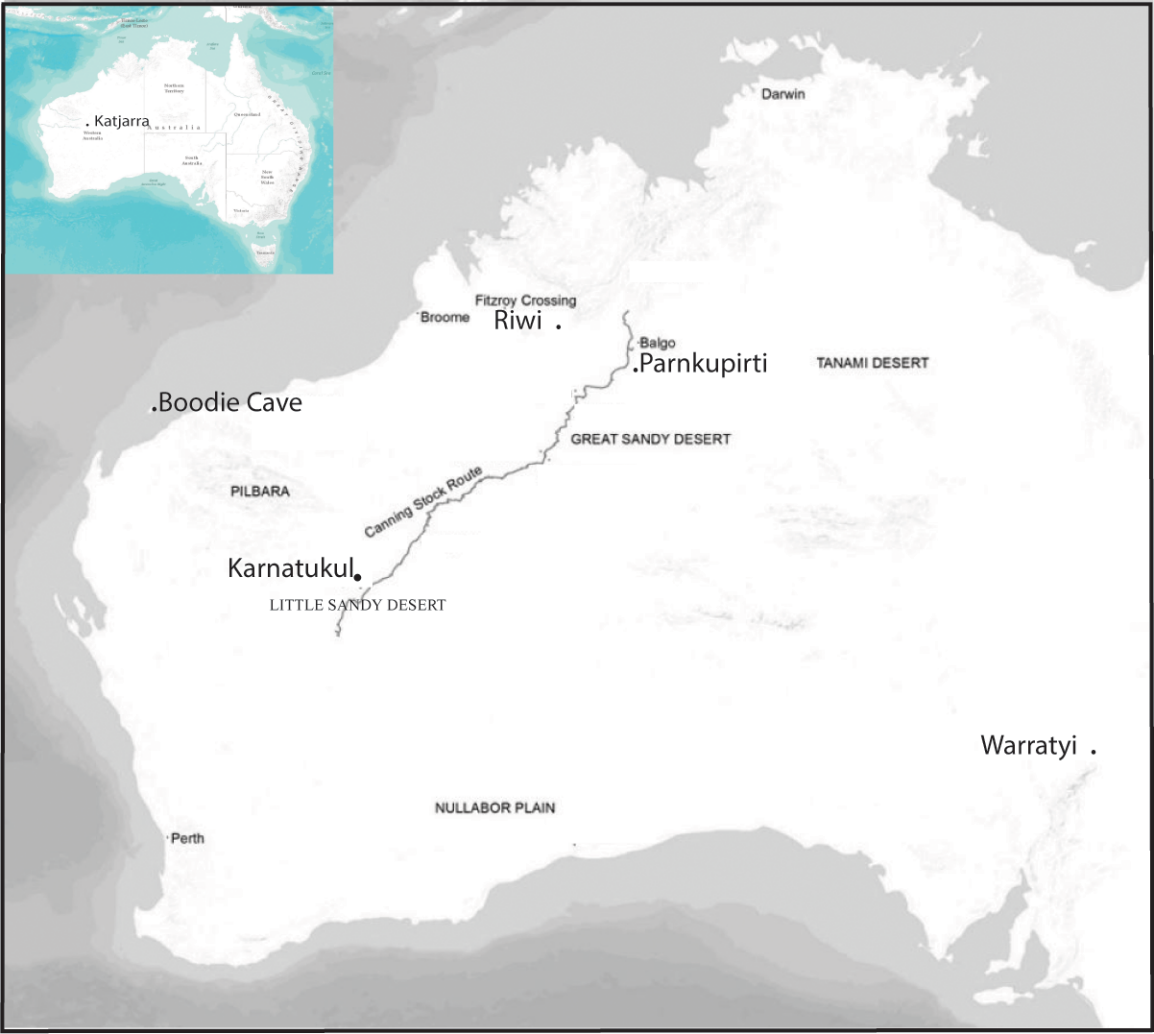
Location of *Karnatukul* in the Western Desert, showing the location of sites named in text.

Re-excavation of the site aimed to contextualise the recent painted art production (Fig 1) and to refine the occupational sequence. Direct-dating of pigments has shown this art production took place between 800–200 years ago [6]. To explore the contemporaneity of rock art production with other occupation evidence we re-carried out targeted excavations of the deposit.

Occupation of Australia is now accepted as having occurred prior to 60,000 years ago [7] with increased evidence for arid-zone occupation prior to 40,000 BP and a number of sites dated to before and during the LGM (for example, [8–15]). *Karnatukul* retains its significant position as the earliest rockshelter to be occupied in the Western Desert, with this excavation demonstrating that this site was first visited by at least 45,190 years ago.

## *Karnatukul*: Site description

*Katjarra* (the Carnarvon Ranges) lies within the Western Desert cultural bloc [2, 16], and *Karnatukul* lies in a broad valley at the western end of *Katjarra*. This quartz-sandstone range comprises an extensive upland on the otherwise low relief sandy plains and dunes of the Little Sandy Desert (Fig 1).

**Fig 2:**
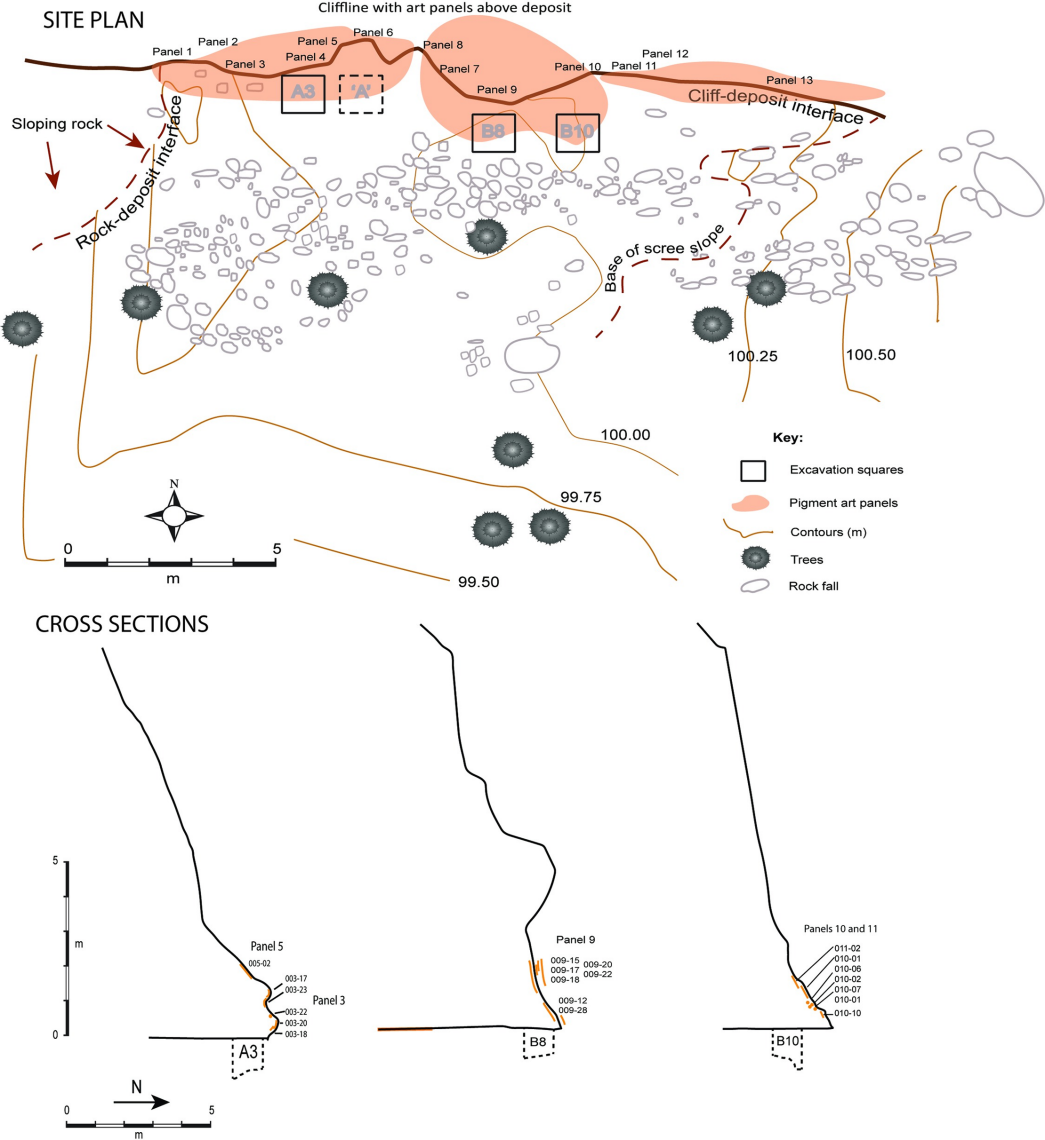
*Karnatukul* site plan and cross sections (original square ‘A’).

**Fig 3:**
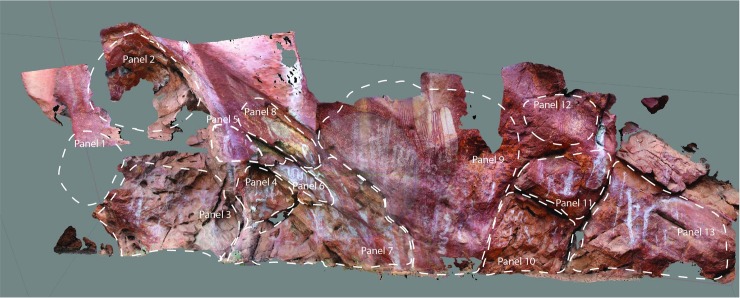
*Karnatukul* showing the nature of the site’s occupation floor and its surrounds during excavation (Photograph Peter Veth).

The shelter is comprised of an overhanging 6m high cliff line on the northern side of a southerly draining valley in the south-western corner of *Katjarra* (Fig 2). The large sandy flat within the small gorge, has a stream channel which discharges on the opposite side from *Karnatukul’s* painted panels. This sandplain–inside the valley and beyond–has many surface grindstones and smaller artefacts, though the number of these has diminished since earlier work in 1994 (Peter Veth, pers. observ. 2005, 2017). The site’s deposit has accumulated at the base of the overhanging cliff line, forming a high, albeit shallow dripline. Only Panels 2–7 are fully protected from water run-off. The majority of sediments are Aeolian, deriving from the surrounding dune fields. The slowly eroding quartz-sandstone geology provides little sedimentary contribution other than *in situ* weathering. Sediments may derive in part from the large fissure in the cliff 20m to the east of the rockshelter, although this is only active after heavy localised rains. The shelter floor falls only 1.0m in elevation over 20m distance (east-west), and is flat within 5m of the shelter’s back wall. The broad open valley has a shallow incised creek line on the opposite side, and is a low-energy geomorphic environment as recorded (by PV) during high rainfall storms 25 years ago. Surface water rose gently within the valley minimally covering the rockshelter floor. Beyond the protected area of deposit, sloping bedrock is exposed a few meters away to the west with continuing roof fall of small blocks and boulders (Fig 2, Fig 3). At the head of the valley the creek line constricts and there are several relatively reliable rock pools, along a mostly pebbly creek line. A boulder here has heavily weathered track engravings, and there are several vertical panels with pigment art. There are no geomorphic processes in this area to provide significant accumulations of sediment.

### The rock art

The rock art at *Karnatukul* was first photographed in 1967, at which time a senior custodian Willy Ward was recorded retouching some of the art [17]. There is also pecked graffiti (SERPENTS GLEN) from the same era by Peter Muir, well-known dogger and author, who chipped his initials or site name in various locations around the Ranges. *Karnatukul* is *Katjarra*’*s* largest rock art assemblage [18]. The current investigation of possible contemporaneity of occupation and art production required detailed analysis of the art assemblage’s superimposition relationships.

The site has 237 recorded motifs, of which 166 can be classified according to the Western Desert motif taxonomy [19]. For the remaining 71 motifs only technique, colour and form were recorded.

**Fig 4:**
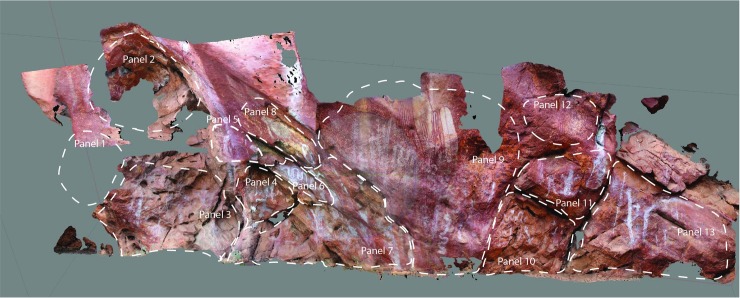
*Karnatukul*’s rock art panels showing panel identification.

**Fig 5:**
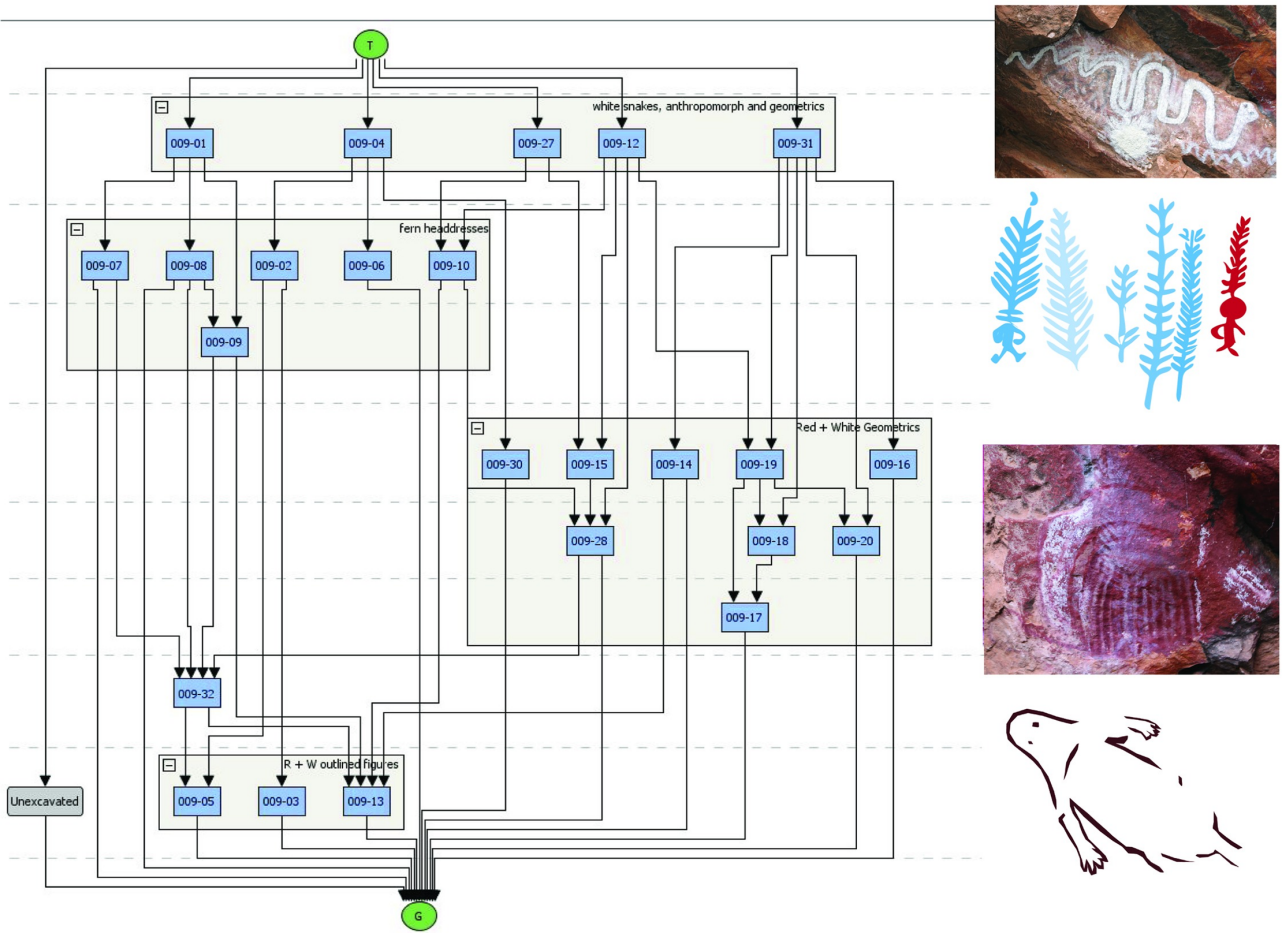
Harris Matrix for Panel 9 showing relationship of the five phases of art production at *Karnatukul*.

The art is found on 13 panels across the site; these loosely defined by breaks in the shelter wall morphology (see Fig 4). Most of the art is pigment (painted, stencilled, drawn) and found between 20cm and 350cm above the current shelter floor. Three very weathered pecked dots (cupules?) and two weathered parallel bars (incised grooves?) are located on sloping bedrock just above the current floor level on Panel 7, which has 63 pigment motifs (of which 23 are recognisable). This Panel is located in a low alcove just above the main occupation floor. Panel 9 is the most visually dominant panel and this has a major bichrome composition of 36 identifiable motifs. Superimpositions are found on all 13 panels, which is indicative of the episodic nature of the art production. Superimposition analysis of the pigment art and its spatial distribution refine a site-specific pigment art chronology (Fig 5; Table 1).

**Table 1:** *Karnatukul*’s pigment art sequence.

Phase	Description	No. of motifs	WD Phase [6
5 (most recent)	Monochrome solid human figures, snakes, phytomoprhs and turtles: mostly white, cream grey (some bichrome), rare late use of yellow. Human figures are mostly between 40-70cm in size; several of these human figures, phytomorphs and snakes have been retouched (in a later episode?) with drawn or painted black charcoal around their outlines.	117	Phases 7, 8
4	Red monochrome linear track and geometric motifs;	27	Phase 7?
3	Monochrome fern headdresses (mostly white: one red and one red + white); with and without human figures attached. Where attached the human figures are linear and small (<30cm); some have associated material culture;	12	Phase 6
2	Red outline and white bichrome outline and linear infill geometrics and attenuated human forms; likely associated with red outline and white infilled snake;	23	Phase 5?
1 (earliest)	Red outline and cream/white solid anthropomorphs with red elements; medium sized (>60cm), located high above the floor (this is possibly contemporaneous with phase 2)	4	Phase 5?

The majority (64%) of the art has been produced during Phase 5: most of this art (89%) is monochrome and this mostly (76%) white and/or cream. The red and white bichrome phases–which, from their composition are interpreted as being contemporaneous, are by contrast predominantly (91%) bichrome. Phase 3 is mostly white monochrome while Phase 4 is entirely red.

Panel 9 is a palimpsest containing all five defined phases, with the genesis of this sequence being the striking red and white bichrome composition (Fig 4). It is likely Phases 1 and 2 were produced contemporaneously [20] and that this red and white bichrome phase included both human forms and geometrics. On Panel 9, however, there is only a single overlap with a barred red and white oval over one of the red outlined human figures (009–14 over 009–13). This provides inconclusive evidence for contemporaneity given that the two subjects are mostly spatially discrete. The nature of the relationship between these earliest motifs is obscured by the overpainting of three subsequent phases. Differential weathering across the panel–largely water damage/development of geo-crusts [21]–also make demonstrating the association of these earlier composition elements more difficult. The Phase 3 white fern headdress figures are clustered to the left to Panel 9 while a single red and white fern is located to the right over several of the barred and bisected oval designs. Two large white convoluted monochrome snakes cover the centre of the panel. The single white human figure with a ‘glove-hand’ headdress and multiple digits on feet and hands is superimposed over the headdress figures.

#### Rock art dating results

The direct-dating of pigment rock art in the Western Desert was a significant outcome of the ARC Canning Stock Route: Rock art and Jukurrpa Project [6]. All *Karnatukul* pigment dates were derived from charcoal pigment art sampled in 2005, after a site visit in 2004 with custodians to seek necessary permissions. Plasma oxidation pre-processing combined with the AMS technique [22] was employed to date the most recent art phase. Ten samples were collected from this site (including associated backgrounds) but only seven (two, backgrounds) had sufficient charcoal to obtain a reliable age determination (Table 2). All the black pigment samples were charcoal (confirmed by magnification of visible wood grains). The significant overlap (at 95% probability) of all of these motifs, suggests episodes of production after 750 calibrated years ago. The grey anthropomorphic figures on Panels 3 and 4 (described as *mamu* by the traditional owners) created from white pigment mixed with charcoal/ash may have been produced slightly later than the spatially discrete black and white phytomorphs (on Panels 11 and 13). Note that use of DStretch [23] has located red pigment beneath mamu 003–16, meaning that background 18b is not technically a background rock sample, but may be affected by a red geometric motif not observed macroscopically in 2005. The background samples in this superimposed site are not considered to be identifying naturally deposited/generated carbon (i.e. sources of natural contamination).

**Table 2:** Motifs sample numbers and age determinations from *Karnatukul* (from 6^.^: Tables 2–4).

Panel Motif ID	Sample No	CAMS ID	Radiocarbon Age (Years BP)	Calibrated Age (cal. BP) (95%)	Description
003–16	18	134002	285 ± 35	450–150 cal. BP	Grey mamu
004–04	19	133998	460 ± 40	550–350 cal. BP	Grey mamu: charcoal drawn outline sampled
004–02	20	136689	795 ± 45	750–550 cal. BP	White phytomorph
011–03	24	130499	745 ± 45	750–550 cal. BP	B+W phytomorph–black sampled
013–01	25	133999	540 ± 80	700–300 cal. BP	W+B phytomorph–black sampled
003-16b	18b	133997	3,190 ± 60	3,550–3,160 cal. BP	Background sample (Phase 4 red?)
004–02	20b	158160	2,050 ± 200	2,500–1,400 cal. BP	Background sample (wall)

## The excavation

Three 1m x 1m squares were excavated at *Karnatukul* (Fig 2), the primary purpose of these being to correlate the art production with occupation evidence. All squares were dug in 2 cm excavation units (XUs) or in stratigraphic units (SUs)–whichever were smaller. Two of these squares (B8 and B10) were placed below Panel 9 (the main rock art composition); the third (square A3) was placed in a more interior location (beneath Panels 3, 4 and 5) west of the original excavation square (see Fig 2). Square A3 was excavated in an effort to replicate the original faunal assemblage retrieved 20 years before [1], as fauna is largely absent from squares B8 and B10. A fourth square was excavated on the sandplain approximately 50m away from the rockshelter to assess the depth of the open sand sheet and occupation evidence (SQ SSA). This was found to be largely a poorly developed and shallow deposit with only a surface manifestation of occupation evidence.

This paper reports on the analysis of all materials recovered from squares B8 and B10. Square A3 contains even higher-density recent archaeological evidence than squares B8 and B10, but also has concomitant evidence for intensive occupation disturbance. Its large lithic assemblage is still being analysed and catalogued as ongoing research. All materials are stored in the secured UWA Archaeology Lab. All analysed material will ultimately be lodged with the West Australian Museum, in accordance with their Guidelines. Our online database (doi:10.4225/23/5ab83d1486aaf) includes the WA Museum tracking number WAM DA-T2018.05.01.

### Sediment and stratigraphy

**Fig 6:**
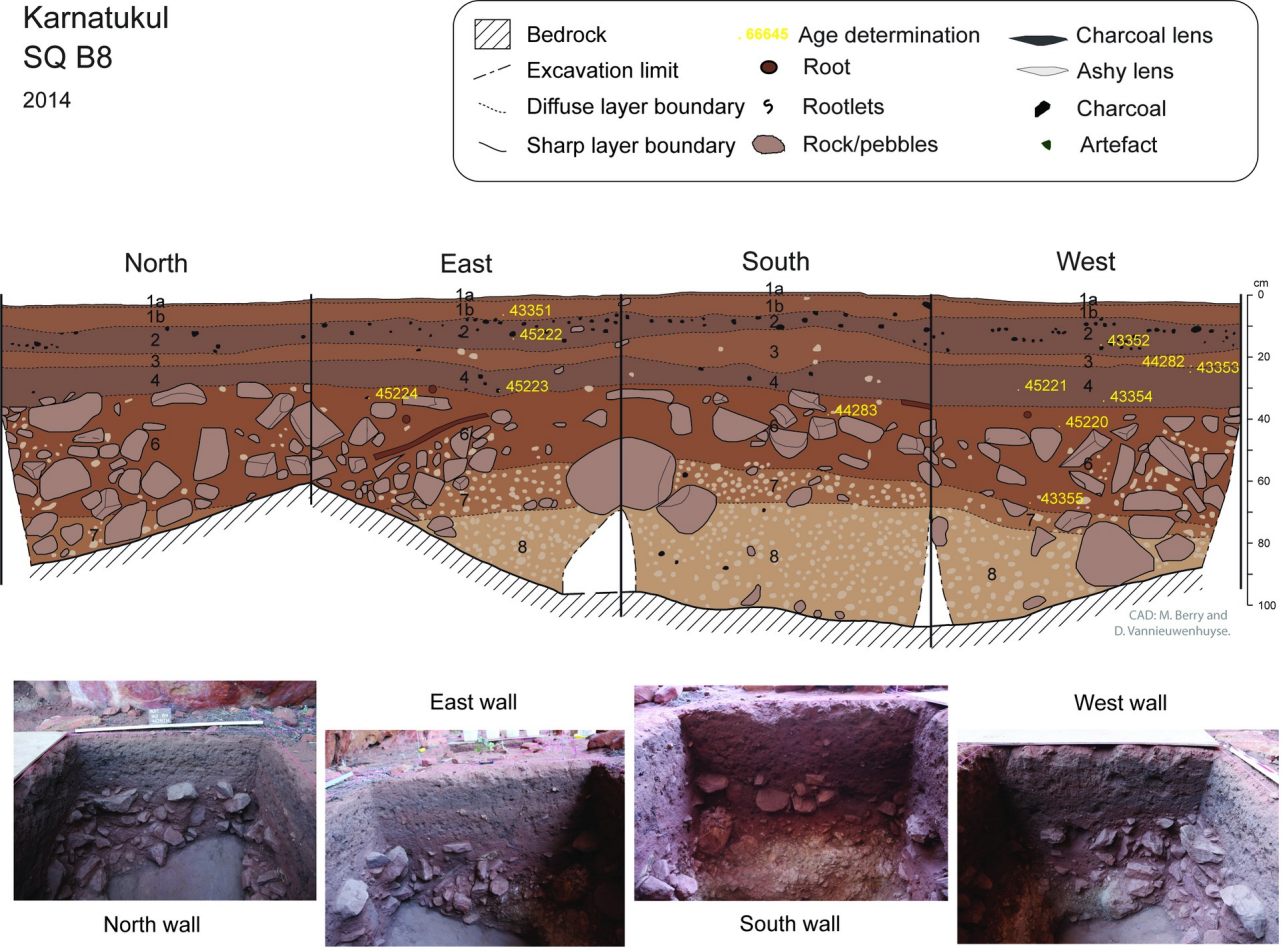
Square B8: Stratigraphic section drawings and end excavation photographs for all four finished sections. Waikato age determination sample points shown in yellow.

**Fig 7:**
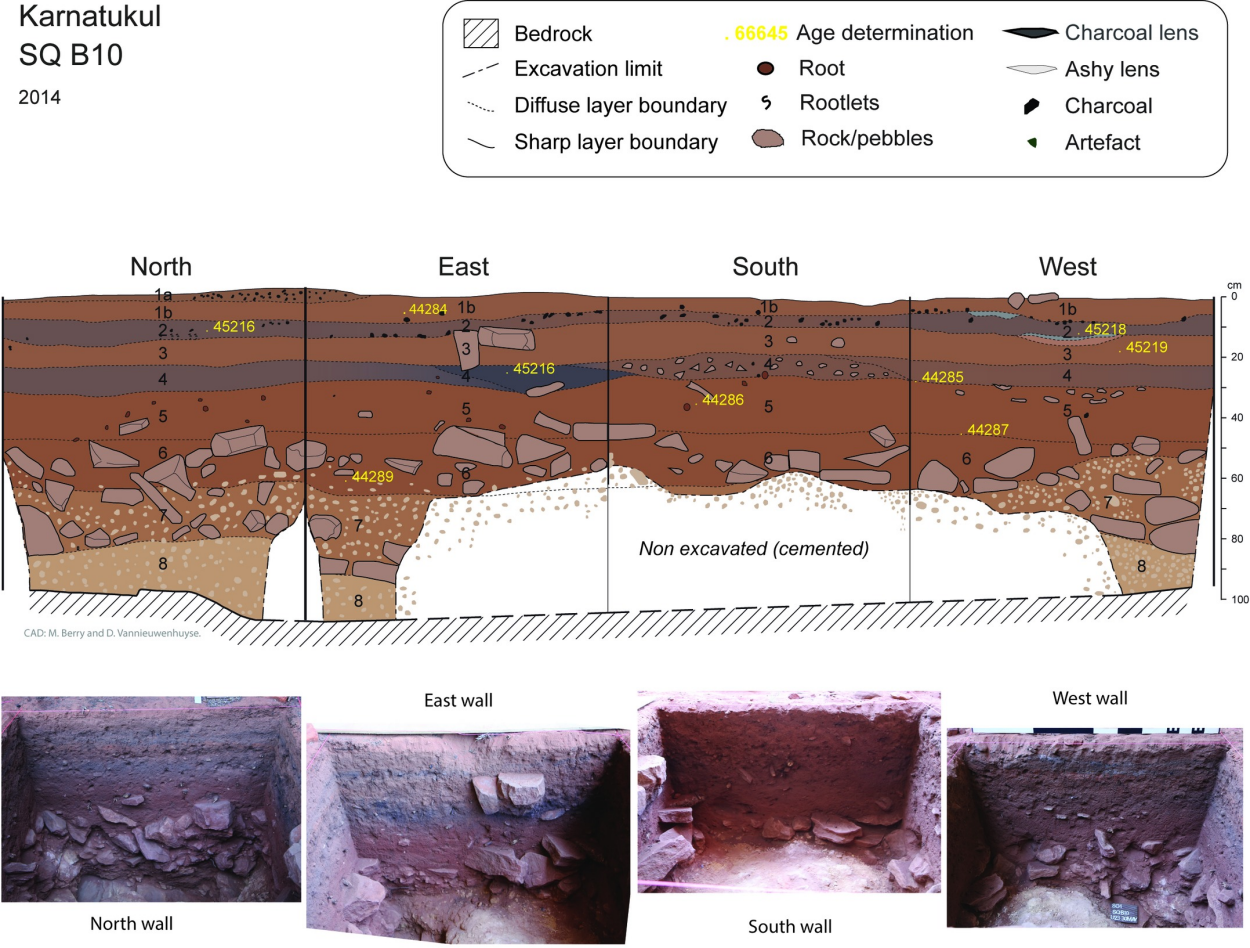
Square B10: Stratigraphic section drawings and excavation photographs for all four finished sections. Waikato age determination sample points shown in yellow.

**Fig 8:**
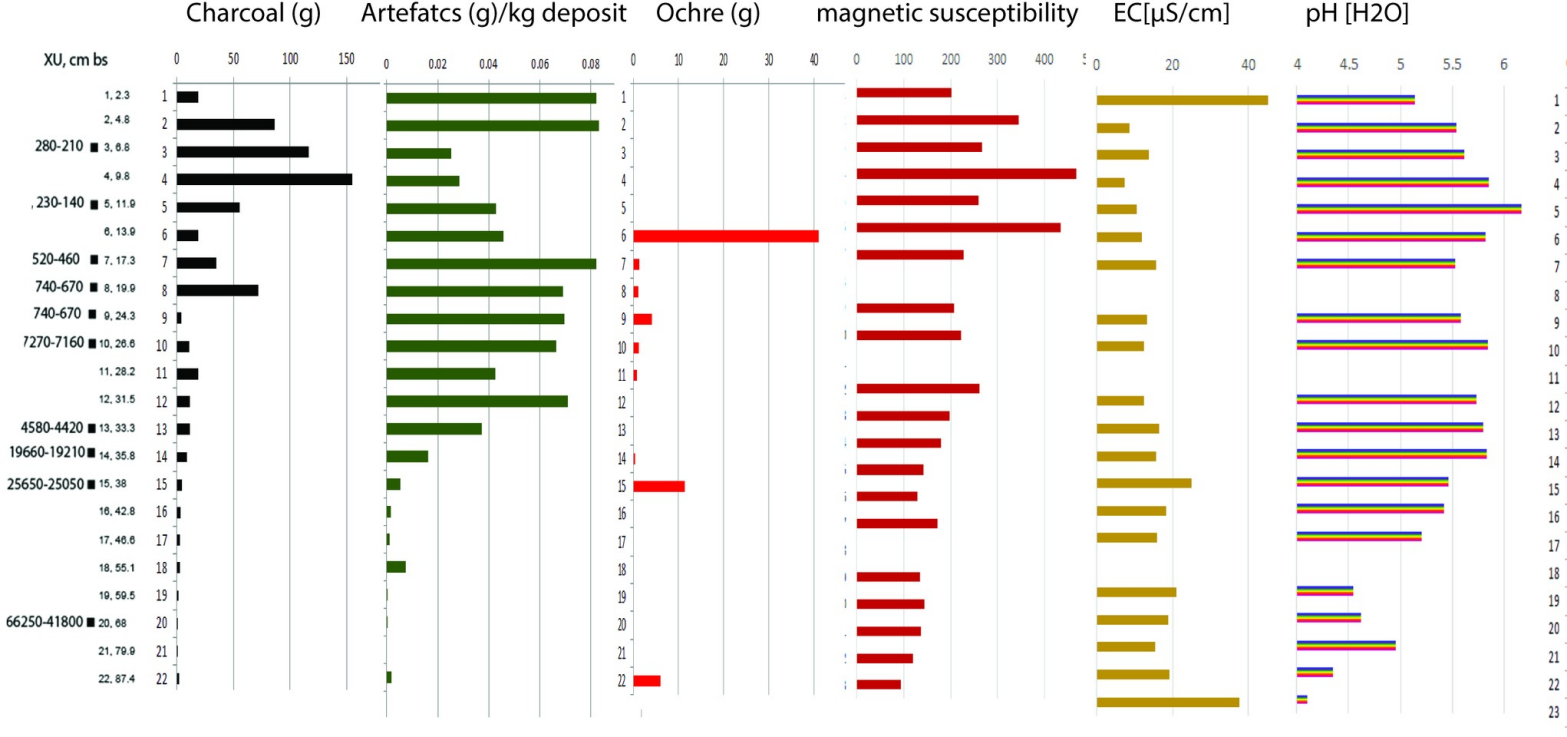
Square B8 correlation of sediment analyses, lithics, pigment and charcoal.

Bedrock was reached at between 80 and 100 cm depth in all three squares (Table 3). The sequence is composed of eight stratigraphic layers mainly defined by changes in colour and presence/absence of rocks or pebbles (Figs 6 and 7). The basal units which were heavily consolidated above sloping bedrock were excavated according to stratigraphy in square B8. In square B10, the highly cemented layers SU8 and SU7 were not fully excavated. Soil acidity (pH) and Munsell colour was recorded during excavation. Sediment samples were collected from each XU and a series of laboratory analyses undertaken, including sediment particle size, magnetic susceptibility, electro-conductivity (Figs 7 and 8).

**Table 3:** Correlation of excavation units (XU), stratigraphic units and analytical units (AU): Squares B8 and B10.

Strat Unit	B8 XUs	B8 AU	B10 XU	B10 AU	Period
1a	1	1	surface	1	Last Millennium
1b	2, 3	1	1, 2, 3	1	
2	4, 5, 6,	1	4, 5, 6	1	
3	7, 8, 9	1	7, 8, 9,	1	
4	10, 11, 12, 13,	2	10, 11,	2	Mid Holocene
5	-		12, 13, 14	3a	Pleistocene-Holocene transition
6	14, 15, 16, 17,	3b	-		LGM
6	18–20		15–21	4	Pleistocene Unit
7	21	4	22	4	
8	22, 23	4	23	4	

### Stratigraphic units

The stratigraphic units can be summarised as:

1a Loose topsoil. Dark reddish brown (5 YR 3/4) coarse and medium sand with some charcoals, twigs and decayed leaves.
1b Reddish brown (5 YR 4/4) coarse and medium sand, some charcoals.
2 Brown to dark brown (7.5 YR 4/4, 7.5 YR 3/4) coarse and medium sand with numerous charcoals, colour varies (darker or greyish >2b) depending on concentration of charcoals and ashes.
3 Reddish-brown (5 YR 4/3) coarse and medium sand.
4 Dark brown (7.5 YR 3/4) coarse and medium sand with abundant charcoals, colour varies depending on concentration of charcoals.
5 Strong brown to dark brown (7.5 YR 4/6, 7.5 YR 3/4) medium and coarse sand without large rocks and presence of small roots, colour varies depending on intensity of downgrading of darker particles from SU4 (sqA3).
6 Strong brown (7.5 YR 4/6) medium and coarse sand with numerous large rocks, presence of roots.
7 Yellowish brown (10 YR 5/4) silty fine sand, with numerous whitish gravels and pebbles, numerous rocks with carbonate crust weathered surfaces, some small roots.
8 Cemented very pale brown (10 YR 7/4) silty fine sand, with numerous whitish gravels and pebbles, bedrock surface is weathered with carbonate crust, some minor roots.

The upper layers (SU1-4) have distinct layering. The surface topsoil is very loose and particularly coarse, probably due to low energy slope wash and/or wind having removed fine particles. The upper layer (particularly SU2 and SU4) are highly enriched in anthropogenic particles such as charcoals and ashes in variable proportion, altering the sediment colours from dark brown to greyish. Some combustion features are visible in sections, as are lenses of ashes (see Figs 5 and 6) and heat-damaged artefacts. These features indicate more intensive periods of recent occupation of the shelter. The visible alternation in this upper sequence between highly enriched anthropogenic residues (SU2 and SU4) and more natural layer (SU1 and SU3) is interpreted as episodic intensive occupation events between 500–700 years ago and then 0–300 years ago interspersed with less intensive occupation.

SU5 and SU6 have similar texture and colour: SU5 is characterized by sandy deposit and the absence of large rocks; SU6 contains some large horizontally bedded blocks indicating an episode of rock fall (see Fig 2).

**Fig 9:**
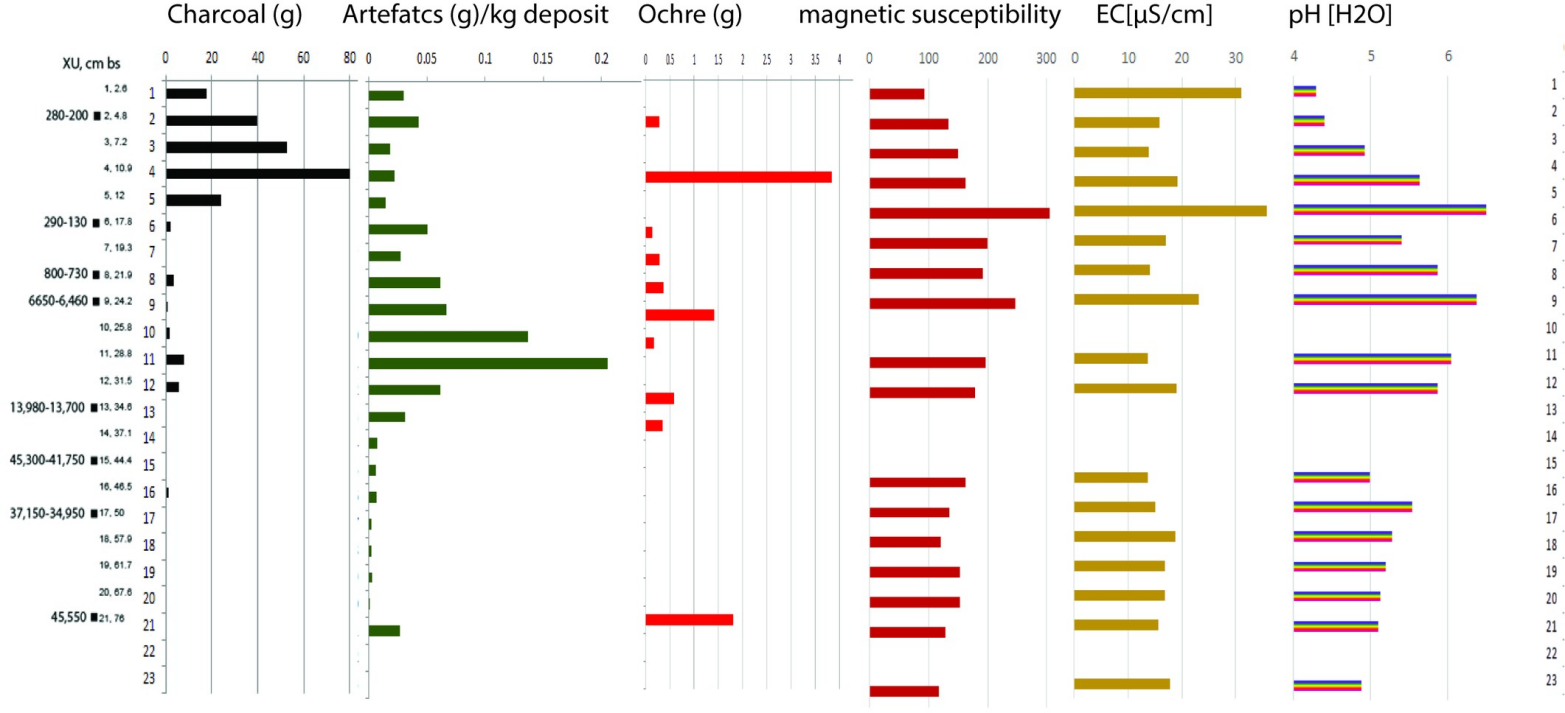
Square B10 correlation of sediment analyses, lithics, pigment and charcoal.

Stratigraphic unit 5 is absent from square B8, indicating different lateral deposition histories within the shelter, even within close proximity (i.e. squares B8 and B10 are separated by one metre). This is confirmed by variations in the cultural sequence (see Figs 8 and 9). This stratigraphic absence is interpreted as site cleaning from the vicinity of B8 at the commencement of the mid-Holocene occupation, although the absence of a sharp boundary means there is no clear stratigraphic evidence for this. Stratigraphic unit 6 also has a few small roots indicating some minor bioturbation of this deposit could be expected.

SU7 and SU8 show intense weathering processes: principally development of secondary carbonates that have cemented the layers in some areas. These layers point to some degree of water stagnation at the bottom of the sequence. SU8 was not encountered during the 1998 excavation [1] likely because of its location on the sloping bedrock.

#### Sediment analysis

Sediment samples were collected from each XU and a series of laboratory analyses undertaken (Figs 7 and 8). A Malvern Mastersizer 2000 (v 5.6) was used to calculate the particle size distribution of sediment samples. Samples were treated with hydrogen peroxide to remove organic matter prior to analysis. The Wentworth-phi scale was used in further analysis [24]. Volumetric magnetic susceptibility was measured using the kappabridge MS2 from Bartington at 0.47 kHz (10^−5^ SI) and EC and pH values were measured in a 1:5 soil to DI-water suspension [25]. This data was used to create a pedological and sedimentological stratigraphy independently of archaeological artefacts.

Sedimentological differences in the profile were analysed by quantifying quotients of the various sand fractions: e.g. coarse (CS), medium (MS) and fine (FS). CS/FS and CS/VFS showed the highest standard deviations (SD) of 0.41 and 0.93, whereas CS/MS and MS/FS yielded SDs of 0.22 and 0.14 respectively. High SD was interpreted as a good indicator for potential stratigraphic changes: thus, we used the quotients of CS/FS and CS/VFS to further explore sedimentological breaks. Potential sedimentological break were suggested where over and underlying samples differed in their quotients of more than half the SD. This is based on the assumption that changes in intensity of occupation and eolian deposition would be reflected by sediment differences [26, 27] and aligned with changes in basic physical and chemical parameters. In the younger sediments magnetic susceptibility seems to be a good indicator of sedimentological breaks with increases between SU3 and SU4 (AU 1b – 2) as well as at SU6 and SU7 (AU3b and AU4). Magnetic susceptibility values decrease in the basal sediments: indicating less frequent fire history (supported by lower charcoal signatures; and see studies by [28]) This aligns with a decrease in pH and generally higher EC values. Deeply acidified sediments at the base of B8 and B10 have pH values <5.5 and <4.4 respectively. In the younger sediments, pH was consistently over 5.5. The opposite trend can be observed in the EC-values. Lower values (10–15 μScm^-1^) are identified in the upper units with slight increases (15–35 μScm^-1^) below. Changes in values of the physical and chemical properties align well with sediment boundaries identified in the field and which have been deployed in the Bayesian model.

#### Anthracology

Anthracology (wood charcoal analysis) provides insights into fire wood collection strategies through time, allowing us to infer not only the mobility of past occupants around a local landscape [29, 30] but providing insights into the nature of the wood materials being dated, as well as potentially enabling palaeo-environmental reconstruction.

Charcoal was collected from all excavation squares. Anthracological protocol was met by capturing both large and small fractions. Distinct hearth features were collected and bagged *in situ*: surrounding sediments were sieved. This strategy provides for representation of both short term collection activities and past vegetation composition. The larger fraction was sorted by hand on site with some flotation undertaken at the camp water tank. The 2mm residue was subjected to flotation in the laboratory, to ensure all botanical remains were collected. A total of 1,835.8 g of charcoal was recovered from the three excavation squares: Square B8 has a well-preserved charcoal assemblage (640.5g).

Here we focus on an upper hearth feature (dated by Wk-43351), and a scattered Pleistocene assemblage (from B8 SU6). This analysis forms part of Byrne’s ongoing doctoral research. Samples submitted for age determinations from both of these features/units were collected *in situ* and not subjected to anthracological identification prior to dating.

**Fig 10:**
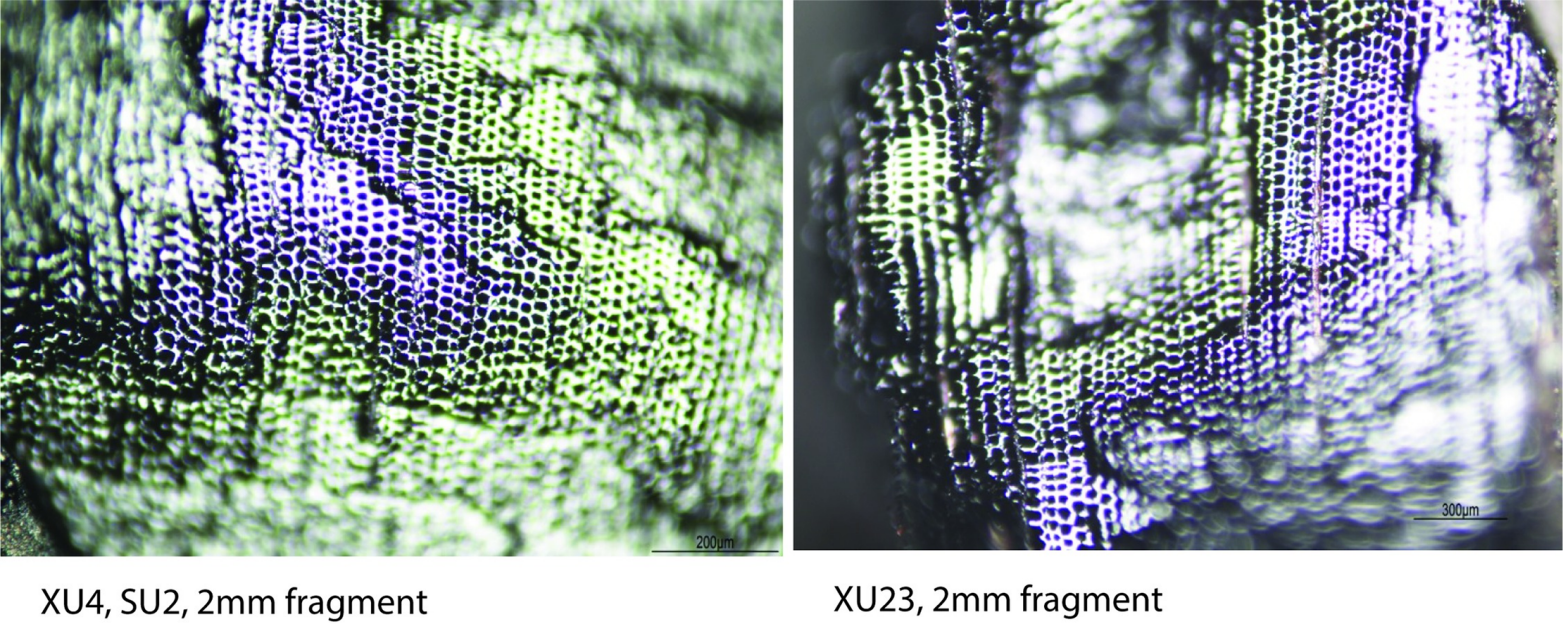
Square B8 (left) Holocene; (right) Pleistocene *Callitris columellaris* (White Cypress Pine): (Transverse Nikon Eclipse LV100ND transmitted light microscope).

**Feature 1.** This condensed hearth feature (dated to the last 160 years: modelled R Date Wk-43351) is characterised by 200 identified fragments (following [31, 32]). White Cypress Pine (Callitris columellaris) is the most frequent species identified (see Table 4, Fig 10) followed by Acacia. Callitris is not known to be a subsistence resource, unlike Acacia, Ficus or Santalum (also identified). Satalum and Ficus both produce edible fruits, not just seeds [33, 34]. Callitris grows in close proximity to the shelter today and has been recorded as providing wood and resin for tool-making and firewood (33:135). The frequency and combination of these taxa indicate that people in the recent past were collecting firewood from nearby vegetation units from the sandstone ranges and rocky side slopes around the shelter today.

**Table 4:** Square B8 charcoal features. Identified plant taxa.

	SU2 (AU1)		SU7 (AU4)	
TAXON IDENTIFIED	# identified	frequency %	# identified	frequency %
CUPRESSACEAE Callitris columellaris	59	29.5%	4	19.0%
FABACEAE Acacia cf adsurgens	4	2.0%	0	0.0%
FABACEAE Acacia cf aneura	3	1.5%	2	9.5%
FABACEAE Acacia cf aptaneura	37	18.5%	2	9.5%
FABACEAE Acacia cf ayersiana	18	9.0%	0	0.0%
FABACEAE Acacia cf minyura	2	1.0%	0	0.0%
FABACEAE Acacia cf pruinocarpa	2	1.0%	0	0.0%
FABACEAE Acacia sp	48	24.0%	7	33.3%
MORACEAE Ficus sp (virens)	2	1.0%	0	0.0%
MYRTACEAE Eucalyptus sp	0	0.0%	1	4.8%
cf MYRTACEAE Eucalyptus sp??	0	0.0%	2	9.5%
SANTALACEAE Santalum cf lanceolatum	1	0.5%	0	0.0%
Unknown (cf AMARANTHACEAE Ptilotus)	1	0.5%	0	0.0%
Unknown (FABACEAE Acacia)	20	10.0%	0	0.0%
Unknown	3	1.5%	3	14.3%
**TOTAL**	**200**	**100%**	**21**	**100.0%**

**Pleistocene layer.** The Pleistocene unit (which began accumulating before 45,190 cal. BP) produced six identifiable taxa from 21 fragments (insufficient for palaeoenvironmental reconstruction). Acacia and Callitris are the dominant species: indicating some continuity in the vegetation communities around the site through time and preferences for these. Several Eucalyptus species identified were not found in the recent hearth. Woods such as these are likely to have derived from stony hills, creek lines and drainage channels, some distance from the rockshelter.

### Dating the sequence

A total of 22 radiocarbon determinations have been returned from squares B8 and B10 (Table 5). In Stratigraphic Units 1–4 charcoal was collected from identified burning features associated with artefacts. Several fragments of charcoal observed in cleaned sections (from SU4 and SU6) were collected from square B8: all other dated charcoal samples from the lower units (SU 4–6) were collected in situ during excavation (Table 5). All dated samples are plotted against their nearest wall at the recorded depth below surface (Figs 6 and 7). Field notes for the site’s earliest dated samples record that charcoal was collected from a “feature” in the western wall at the base of B8 (in XU 20: Wk-43355); while the charcoal sample collected from the base of B10 (in XU21: Wk-44289) was located “above the travertine and below a piece of roof fall”. No charcoal was retrieved from SU 7 or SU 8, probably a result of the increasingly acidified deposit with depth.

**Table 5:** Radiocarbon dates from *Karnatukul* squares B8 and B10.

Wk-Code	Field ID	Collection method	Depth below surface	SU	δ^13^C	^14^C date (BP)	Unmodelled calibrated age (Cal. BP, 95% probability range)
44284	B10-XU2-4	Excavation in situ	3.5	1b	-23.5 ± 0.3#	169 ± 21	280–0
43351	B8-XU3-1	Excavation in situ	5	1b	*	163 ± 20	280–0
45219	B10-XU4-3	Excavation in situ	10	2	*	132 ± 15	260–0
45218	B10-XU6-3	Excavation in situ	10.6	2	*	185 ± 15	290–0
45222	B8-XU5-4	Excavation in situ	14	2	*	207 ± 16	290–140
45217	B10-XU8-4	Excavation in situ	14.8	3	*	915 ± 16	800–730
43352	B8-XU7-5	Excavation in situ	16	2	*	469 ± 20	520–460
44285	B10-XU9-3	Excavation in situ	21.3	4	-24.0 ± 0.3#	5,795 ± 23	6650–6460
44282	B8-XU8-7	Excavation in situ	21.5	3	-24.4 ± 0.3#	839 ± 20	740–670
43353	B8-XU9-11	Excavation in situ	23	3	*	840 ± 20	740–670
45216	B10-XU11-1	Excavation in situ	25.1	hearth 3/4	*	391 ± 17	500–320
45221	B8-XU10-3	Excavation in situ	27	4	*	6,332 ± 16	7,270–7,160
44286	B10-XU13-1	Excavation in situ	32	5	-23.4 ± 0.3#	11,989 ± 36	13,980–13,580
45224	B8 CH04 wall	Wall section	32	6	*	1,019 ± 15	930–800
45223	B8 CH05 wall	Wall section	26	4	*	2,949 ± 15	3160–2950
43354	B8-XU13-1	Excavation in situ	33	4	*	4,071 ± 20	4,580–4,420
45220	B8-XU14-5	Excavation in situ	36.5	6	*	16,160 ± 70	19,660–19,210
44283	B8-XU15-6	Excavation in situ	34.5	6	-23.6 ± 0.2#	21,057 ± 106	25,450–25,080
44287	B10-XU15-2	Excavation in situ	40.6	6	-23.5 ± 0.3#	39,345 ± 1082	45,300–41,790
44288	B10-XU17-1	Excavation in situ	44–47	6	-23.9 ± 0.3#	32,104 ± 425	37,110–34,960
43355	B8-XU20-3	Excavation in situ	67	6	*	47,039 ± 3737	>49,320
44289	B10-XU21-1	Excavation in situ	68.3	6	-25.2 ± 0.2#	45,929 ± 2716	>45,590

All charcoal samples were sent to Waikato, NZ, where they were pre-treated by washing in hot HCl, rinsed and treated with multiple hot NaOH washes. The NaOH insoluble fraction was treated with hot HCl, filtered, rinsed and dried. Despite their relative proximity squares B8 and B10 show internal variation.

* ^13^C measured by AMS. Result not reported

#^13^C measured by CRDS [38].

NB the EDM data point for sample B10-XU17-1 was erroneous, so the exact provenance of this age determination is not known.

These age determinations have refined the occupation sequence [1] and identified occupation from before 45,190 cal. BP; an LGM occupation signal; a Late Pleistocene/Holocene transition; and a Holocene sequence with defined occupation pulses during the last millennium. There are very few anomalies, and these may be explained by localised bioturbation during the most intensive occupation phases. One hearth (Wk-45216) may well be a dug combustion feature [35], which would explain the inversion of this date. As the lower stratigraphic units were heavily cemented and included a high proportion of roof-fall rocks and boulders, no sediment samples could be collected for OSL dating. No charcoal was observed or recovered from these layers. Radiocarbon dates have been calibrated using Oxcal v.4.2 [36] and the SHCal13 [37, 38] calibration curve.

### Bayesian analysis

To provide the most probable chronology for *Karnatukul*, we conducted a Bayesian Sequence Analysis in OxCal 4.2 [36, 39]. To assess the likelihood of any one sample being an outlier, a General t-type Outlier Model is inset into the Sequence [40]. All dates are assigned a prior outlier probability of 0.05. This allows outliers to be either too young or too old, and down weighs their influence in the model. A stratigraphic Bayesian analyses was conducted for *Karnatukul* where all the radiocarbon dates were ordered on the basis of stratigraphic and sedimentological evidence (see above; Fig 9 and Table 6). These are grouped according to six dated stratigraphic phases (SU1 through to SU6); to signify the short micro-hiatuses between SU1 to SU3 we have used contiguous boundaries [see 36] while SU4/SU3, SU5/SU4 and SU6/SU5 are interpreted as sequential (i.e. discontinuous).

**Table 6:** Results of the ‘stratigraphic’ Bayesian analysis for *Karnatukul* where “OP” stands for outlier posterior and ‘C’ for convergence.

Name	Modelled (68%)		Modelled (95%)		Summary Statistics	Indices	
	From	To	From	To	Median	OP	C
Boundary SU1 top	110	-60	150	-260	0		99.7
**Phase SU1b**							
R_Date Wk-44284	120	-10	160	-10	80	99.6	99.3
R_Date Wk-43351	120	-10	160	-10	70	99.5	99.3
Boundary SU2/SU1	180	20	240	0	110		98.3
**Phase SU2**							
R_Date Wk-45219	260	110	270	40	230	98.9	99.2
R_Date Wk-45218	280	140	290	70	220	99.6	98.4
R_Date Wk-45222	290	160	290	140	200	99.6	98.3
R_Date Wk-43352	520	330	520	110	350	77.4	52.3
Boundary SU3/SU2	710	260	720	230	420		46.7
**Phase SU3**							
R_Date Wk-45216	770	330	860	320	470	62.1	40.1
R_Date Wk-45217	780	740	800	730	760	99.6	99.4
R_Date Wk-44282	730	680	740	670	710	99.9	99.4
R_Date Wk-43353	730	680	740	670	710	99.8	99.5
Boundary SU3 bottom	920	740	1340	730	840		96.3
Boundary SU4 top	3050	1890	3080	1020	2330		99.8
**Phase SU4**							
R_Date Wk-44285	6630	6490	6650	6450	6550	99.2	99.3
R_Date Wk-45221	7260	7170	7270	7160	7220	99.5	99.1
R_Date Wk-45223	3110	2970	3160	2960	3040	99.5	99.2
R_Date Wk-43354	4530	4430	4780	4410	4480	97.9	99.2
Boundary SU4 bottom	8830	7190	11340	7170	8160		99.4
Boundary SU5 top	13850	11310	13950	8590	12470		99.0
**Phase SU5**							
R_Date Wk-44286	13930	13710	13990	13570	13780	96.9	99.7
Boundary SU5 bottom	15670	13730	17970	13640	14840		99.1
Boundary SU6 top	19460	16980	19560	14880	17900		98.6
**Phase SU6**							
R_Date Wk-45220	19590	19340	19690	19200	19460	98.3	96.0
R_Date Wk-45224	51930	18370	51950	18270	31060		
R_Date Wk-44283	25520	25220	25680	25030	25370	96.9	95.4
R_Date Wk-44287	44190	42310	45570	41650	43330	95.3	94.4
R_Date Wk-44288	36460	35430	37410	34880	35980	95.5	94.8
R_Date Wk-43355	49980	46740	50010	44990	47860	95.2	90.7
R_Date Wk-44289	49970	46580	50010	45190	47830	95.3	91.1
Boundary SU6 bottom	53860	47950	60700	46380	51320		22.9

This model finds 3 major (>20%) outliers; Wk-45224 (100%), Wk-45216 (38%) and Wk-43352 (26%) (see Table 6). The model convergence values generated by the OxCal MCMC algorithms were also low for the SU3/SU2 boundary (46.7%), the Pleistocene boundary (22.9%), and for 6 individual dates. These low values (<95%) indicate many different incompatible solutions to the model [36]. Wk-45224 is a later Holocene date clustered with mid-Holocene dates and is likely intrusive but is not included in the model calculations 100% of the time. Wk-45216 (an intrusive late Holocene hearth at the interface of SU3/SU4) and Wk-43352, however, are included 37% and 27% of the time. These anomalous dates most likely represent short term activity elsewhere in the shelter that is not fully developed in the stratigraphic units of B8 and B10: ongoing analysis of square A3 may resolve this interpretation.

**Fig 11:**
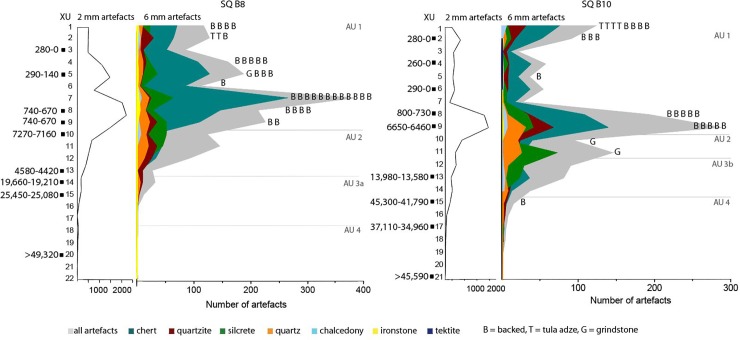
Bayesian sequence model results for the four major occupational phases at *Karnatukul*. 68% and 95% error margins are indicated by bars under each posterior age distribution.

It is worth noting that Wk-44289, Wk-43355 and Wk-45224 produce results that are close to the limits of radiocarbon detection. Any dates which return negative values (SU1b) should be read as modern. This model indicates that the deposit began accumulating prior to 46,380 cal. BP. The chronologies for each strata are shown (Table 6, Fig 11).

## The cultural assemblage

Stone artefacts, charcoal, pigment fragments and a small faunal assemblage were retrieved from the excavations. No sterile deposits were encountered during the current excavations, although several occupational disconformities are indicated by age determinations and the Bayesian analysis.

### Stone artefact analysis

An exceptionally rich assemblage of almost 25,000 artefacts was retrieved from squares B8 and B10 [3,621 stone artefacts (5.98 kg) in the 6 mm sieve fraction; 21,375 artefacts (1.01 kg) from the 2 mm sieve]. Artefacts were identified and categorised following Veth et al. [10] for comparative purposes. Only the artefacts larger than 6mm were analysed in detail. Micro-debitage (the 2mm fraction) has been counted and weighed and classified according to raw material.

#### Raw material use and technological strategies

**Fig 12:**
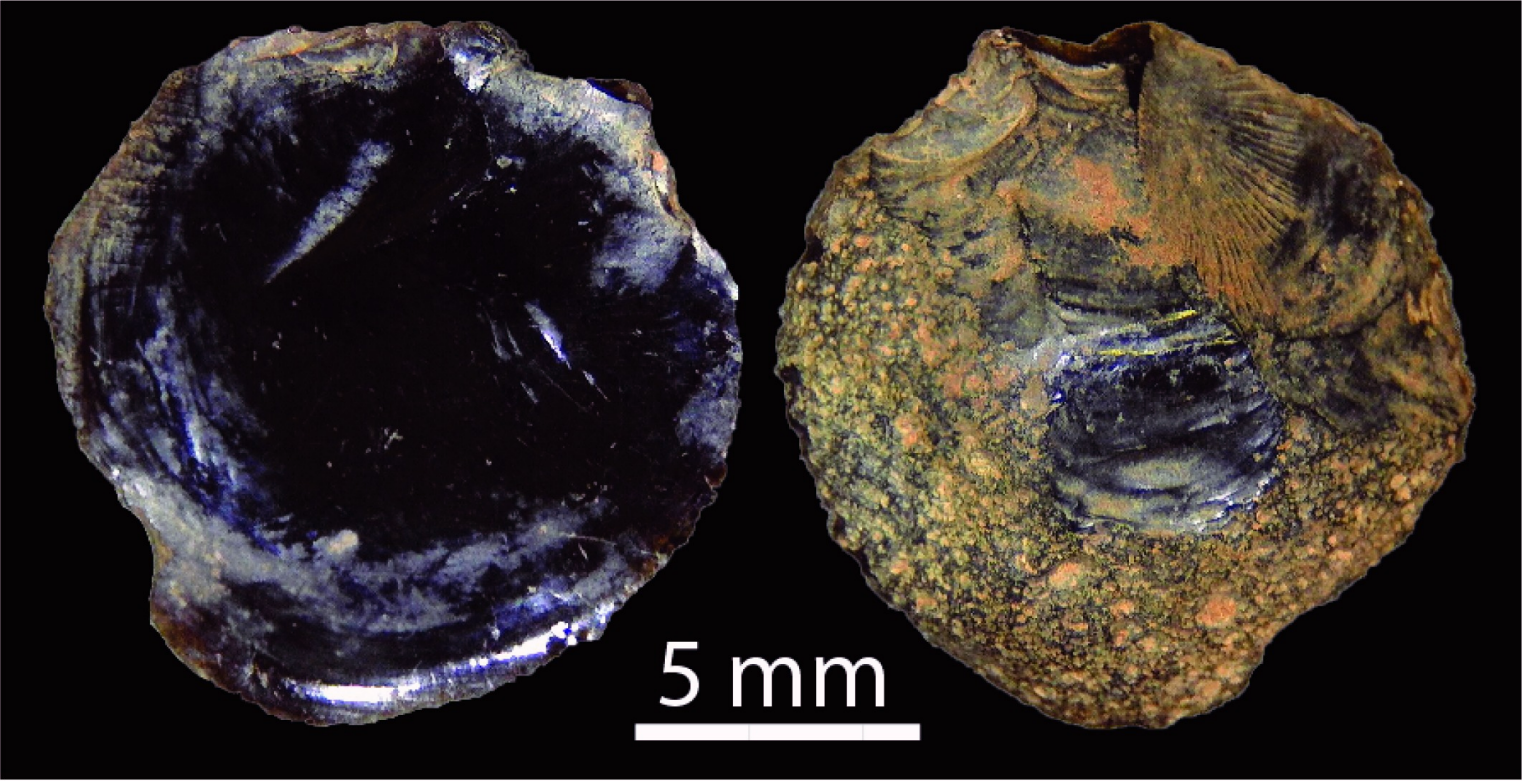
(l) ventral and (r) dorsal surfaces of tektite flake showing pitted dorsal cortex (SG1B1002007).

Over half the assemblage is made on chert (Table 7). Other raw material classes include chalcedony, quartz, quartzite, silcrete, ironstone and (rare) tektite. Tektites are small round objects formed during meteorite or comet impacts: these fall back to the earth’s surface as glass nodules [41–43]. This material is distinguishable from bottle glass because of its distinctive pitted cortex (Fig 12). Tektite is ethnographically recorded as having ideological significance as well as functional use as a stone source (for example, [44–46]). The two small tektite flakes identified at *Karnatukul* were discarded in square B10 during the last 300 years. The earlier excavation [1] located glass artefacts in the top stratigraphic unit. No post-contact materials were located during the current investigation: it is possible that the earlier identified glass artefacts were in fact tektite flakes.

**Table 7:** Squares B8 and B10 6 mm artefact assemblage composition (n = 3,622).

Material	Unmodified complete flake	Debitage	Core/core fragment	Ground fragment	Tula adze/slug	Backed artefact	Other retouch/ usewear	TOTAL
**Chalcedony**	29	41	1		1	1	9	82 (2.3%)
**Chert**	605	1113	12		5	46	137	1918 (53%)
**Ironstone**	10	23					3	36 (1%)
**Quartz**	46	250	10			1	6	313 (8.6%)
**Quartzite**	166	403	4	3		1	15	592 (16.3%)
**Silcrete**	242	400	4			6	26	678 (18.7%)
**Tektite**	2							2 (0.1%)
**TOTAL**	1100 (30.4%)	2230 (61.6%)	31 (0.9%)	3 (0.1%)	6 (0.2%)	55 (1.6%)	196 (5.4%)	3621

Local tool-stone sources around *Katjarra* are currently unmapped but were probably located at least a few kilometres from the rockshelter within the local upland catchment: only low-quality cobbles and gravels (coarse-grained with unpredictable fracturing quality) were noted in the adjacent valley flats (Ditchfield, personal observation, 2014).

Debitage (broken flakes) contributes over half the *Karnatukul* stone assemblage (Table 7). Unmodified complete flakes are the next most common artefact type. Cores are under-represented at the site. Of the 31 cores and core fragments recorded, 12 are made on chert, ten on quartz, and the remainder on quartzite, silcrete, and chalcedony. Only two complete cores retain any cortex and all have at least two negative flake scars (Table 8). There are, however, clear differences in core reduction strategies between materials. Quartz cores are notably smaller than cores made on other materials and three of these cores exhibit features associated with bipolar reduction [47, 48]. This reduction technique results in high frequencies of shatter: reflected by the markedly higher proportion of quartz debitage (80.1%) compared to other materials. All cores indicate that care was taken to prepare platforms for flake removals and that these cores were rotated multiple times to extend the use-life of the material.

**Table 8:** Summary of complete core characteristics (n = 22).

Material (# of cores)	Chalcedony (n = 1)	Chert (n = 8)	Quartz (n = 7)	Quartzite (n = 2)	Silcrete (n = 4)
**Mass (g)**	10.41	12.3 ± 19.1	4.8 ± 14.8	17.6	31.7 ± 25.8
**Maximum dimension (mm)**	34	33 ± 13.3	27 ± 12.5	35	48.5 ± 14.5
**Platform preparation (#, %)**	1 (100%)	5 (62.5%)	1 (14.3%)	2 (100%)	2 (50%)
**Cortex (#, %)**	0	2 (25%)	0	0	0
**Core rotation (#, %)**	0	6 (75%)	3 (42.9%)	0	1 (25%)
**Number of scars**	10	5.5 ± 4.3	3 ± 2.5	3	4 ± 4.3

Each cell contains median and interquartile range unless otherwise stated.

A relatively high proportion of artefacts (n = 201, 5.5%) exhibit evidence for deliberate edge resharpening. Macroscopic edge damage was identified on a further 56 specimens (1.5%) which may be attributed to use. Most of the retouched artefacts (70%) are made on chert, with the remainder made on silcrete (14%), quartzite (8%) chalcedony (4%), quartz (2.5%) and ironstone (1.5%).

The assemblage includes 55 backed artefacts and six tula adze slugs [49]. Chert was the preferred material for backed artefact manufacture (n = 46). Scrapers and notched tools (n = 11) were also recorded. Chert flakes selected for resharpening and use have a significantly higher median mass (n = 61, 1.7 ± 2.8g) than unmodified chert flake blanks (n = 605, 0.4 ± 0.5, U = 5138.5, Z = -9297, p = 0.000), demonstrating the selection of larger chert flake blanks for tool use. Larger silcrete and quartzite flakes also appear to have been selected for tool use. Most tools were not exhausted when they were discarded.

#### Unmodified complete flakes

The size, shape, platform and dorsal attributes on 1,100 unmodified complete flakes (6 mm sieve fraction) were further examined to understand patterns of reduction (Table 9). Large interquartile ranges for mass and estimated surface area on all materials reflect the great variety in flake sizes. Despite this variability, many flakes are small: one third of chert artefacts (n = 5,381; 35.5%) have a maximum dimension less than 5 mm and 89.1% (n = 13,531) less than 10 mm. Many of these small flakes probably derive from tool manufacture and maintenance. A lower density of silcrete tools and micro-debitage suggests that this material was not as intensively reduced. Low proportions of cortex were recorded across all materials. If it is assumed that materials were knapped from cortical nodules, then this suggests that all materials were either bought into the site at a later stage of reduction, or (though less likely), that larger cortical flakes and cores were removed from the site for anticipated use elsewhere in the landscape.

**Table 9:** Summary characteristics of unmodified complete flakes (n = 1,100)*.

Material (# of flakes)	Chalcedony (n = 29)	Chert (n = 605)	Ironstone (n = 10)	Quartz (n = 46)	Quartzite (n = 166)	Silcrete (n = 242)
**Mass (g)**	0.4 ± 0.5	0.4 ± 0.5	0.6 ± 0.9	0.7 ± 1.3	0.7 ± 1.8	0.7 ± 1.2
**Surface area estimate**	130 ± 48	130 ± 108	173.5 ± 107	146.5 ± 152	181 ± 227.3	176 ± 180.8
**Elongation index**	1.1 ± 0.6	1.1 ± 0.7	1.2 ± 0.7	1.1 ± 0.6	1.1 ± 0.6	1.1 ± 0.6
**Overhang removal (#,%)**	15 (46.9%) n = 32	345 (44.7%) n = 771	4 (28.6%) n = 14	23 (36.5%) n = 63	80 (36.4%) n = 220	101 (33.4%) n = 302
**Number of single scar platforms (#, %)**	25 (78.1%) n = 32	581 (75.4%) n = 771	12 (85.7%) n = 14	49 (77.8%) n = 63	182 (83.1%) n = 220	243 (80.7%) n = 302
**Number of dorsal scars**	3 ± 1	3 ± 2	3 ± 2.5	3 ± 1	3 ± 1	3 ± 1
**Flakes with cortex (#, %)**	5 (17.2%)	68 (11.2%)	2 (20%)	6 (13%)	17 (10.2%)	15 (6.2%)
**Feather terminations (#, %)**	27 (67.5%) n = 40	664 (75.6%) n = 878	10 (83.3%) n = 12	60 (76.9%) n = 78	191 (77.3%) n = 247	246 (75.2%) n = 327

*Artefacts included in overhang removal and feather terminations categories include transverse flakes which retained these attributes. Each cell contains median and interquartile range unless otherwise stated.

Platform morphology provides insights into preparatory behaviours that may indicate material conservation [50]. Cortical platforms are rare on all materials (1.8%). While plain platforms are the most common across all materials (Table 9), chert flakes have the highest proportion of platforms with multiple flake scars (n = 66, 8.6%). This type of platform can indicate effort to prepare platforms for more predictable flake removals [51]. More chert and chalcedony flakes have overhang removal (platform preparation) than other materials. This suggests more effort to reduce and conserve chert and chalcedony. However, chi-square tests only found a significant result between overhang removal proportions on chert and silcrete flakes (χ2 (1) = 10.954, p = <0.01). The predominance of small flakes with multiple dorsal scars and absence of cortex suggests that all materials were in a later stage of reduction when they were discarded. This type of economising is consistent with the proposal that prepared nodules were carried into the site from further afield within the landscape.

## Changes in stone artefact assemblages over time

**Fig 13:**
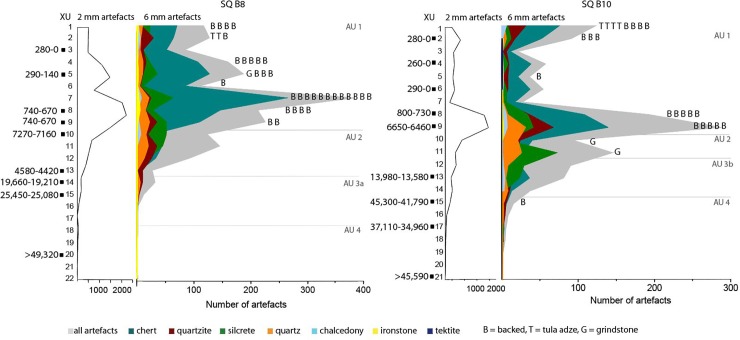
Numbers of discarded artefacts per excavation unit: Squares B8 and B10.

The *Karnatukul* assemblage was divided into five analytical units to examine chronological changes in raw material use and technological strategies (Table 10). These units are interpreted using the Bayesian-modelled stratigraphic units. Despite their proximity, squares B8 and B10 reveal spatial variation in their chronological sequences: e.g. the peak in artefact discard in the last millennium in square B8 is less pronounced in square B10. Discard rates show distinct patterning through time (Fig 13) with similar patterning found in both the 2 mm and 6 mm artefact fractions.

**Table 10:** *Karnatukul* analytical units: Date ranges based on summary statistics (Table 6). Temporal analysis excludes 45 artefacts from provenanced wall clean units.

AU	XUs B8	XUs B10	6 mm total lithics	2 mm total lithics	lithics/kg sediment	Date ranges expressed as summary statistics (ka)
**1**	1–9	1–9	2654	16,899	32	840–0
**2**	10–13	10–11	625	2824	18	8,160–2,330
**3a**		12–14	208	946	8	14,840–12,470
**3b**	14–17		48	322	5	25,370–17,900
**4**	18-base	15—base	41	384	2	51,320–35,980

The assemblage from B10 XU9 (associated with the 6,650–6,450 cal. BP date: Wk-44285) was included in AU1 based on its multiple backed artefacts. We interpret the anomalous date for this archaeological assemblage (Wk-44285) as the result of bioturbation at the base of SU4 at the commencement of intensive occupation during the last millennium. This is supported by the other outlier date (Wk-45216) from a hearth excavated into SU4. There is no Pleistocene-Holocene transition represented in square B8, as confirmed by the absence of SU5 in that square (cf. Figs 6 and 7; Table 3). The LGM signature is found most clearly in B8 SU6. The modelled date range for this analytical unit (Table 10) derives from the two determinations (Wk-45220 and Wk-44283) and the modelled median statistic for the top of SU6 (Table 6). The Pleistocene unit prior to the LGM is replicated in both squares to their bases (note that Stratigraphic Units 7 and 8 were only partially excavated in square B10). The modelled date range for the Pleistocene occupation is based on the modelled lower boundary for SU6 and includes all the Pleistocene dates prior to the LGM (cf. Tables 6 and 10).

### Pleistocene stone assemblages

The Pleistocene assemblage indicates a preference for good quality chert (n = 425, 54.8%), although a range of materials were reduced on-site. Raw material use shifts significantly between the pre-glacial (AU4) and the glacial (AU3a) assemblages (χ2 (4) = 27.92, p = <0.05), as proportions of chert (AU4: 54.8% AU3a: 42.4%) and quartzite (AU4: 24.7% AU3b: 21.4%) decline through time and use of quartz (AU4: 14.1% AU3b: 21.9%) and silcrete (AU4: 4.5% AU3a: 11.9%) increases. This suggests that during the LGM, people were selecting more readily available materials from the adjacent Ranges with reliable water, high ecotonal diversity and other resource suites of economic importance. Increasing focus on local uplands during glacial aridity has been identified at other Australian arid sites occupied during the LGM [9, 12, 52], but not, until now, in the sandy deserts. Raw material proportions remain constant through the terminal Pleistocene (AU3a:AU3b χ2 (4) = 1.67, p = 0.796).

**Fig 14:**
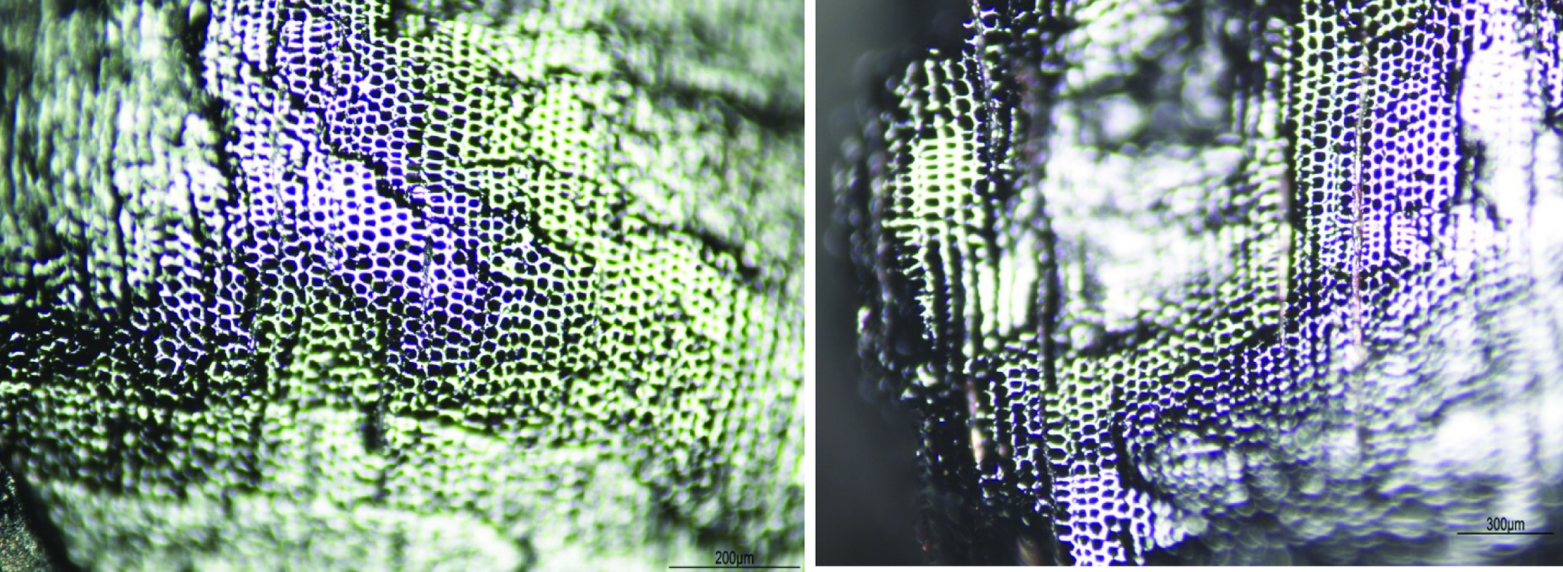
Views of l) ventral and (r) dorsal surfaces, chemically altered, of ironstone scraper (artefact B0822002).

The 6mm *Karnatukul* Pleistocene assemblage (7.8%, n = 297) is mostly comprised of flake blanks (n = 110, 37%) and debitage (n = 181, 60.9%). Only two cores were discarded in this earlier time frame: a rotated quartz core (from B8 XU15) and a quartzite core fragment (from B10 XU12). Four retouched artefacts were identified, including a backed artefact, a scraper and two amorphous retouched chert pieces. The earliest tool recovered from the pre-glacial deposit (in SQ B8 XU22) is a retouched ironstone scraper (Fig 14). This small heavily weathered artefact (located two XU’s below Wk-43355: 47,860 cal. BP, median age) is broken (max. dimension 16 mm) with discontinuous retouch along steep-edge margins. Retouch scars are short, steep and stepped, with macroscopic edge damage evidenced by chattering and small step fractures. Due to extensive water polishing, the type and extent of the break is not clear. However, given its size, use-wear and the type of retouch it has most likely been snapped while being used in a haft (microscopic analysis was undertaken by Richard Fullagar).

**Fig 15:**
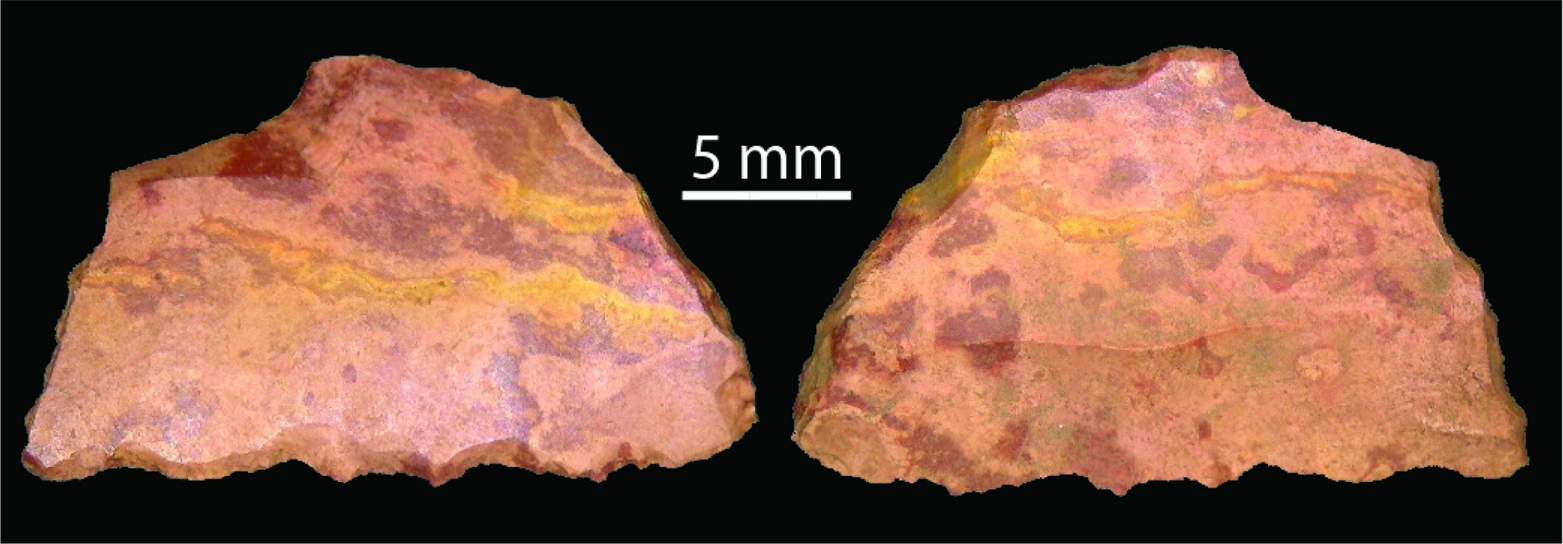
Pleistocene backed artefact B1015001: (l) ventral and (r) dorsal surfaces.

The Pleistocene assemblage includes a geometric backed artefact (measuring 22 x 13.1 x 3.7mm) made on pink veined chert (Fig 15). It has regular continuous retouch as well as use-wear on the chord, mostly on the ventral surface. The backing is discontinuous and mostly unidirectional and includes bending, some feather, step and five points where the Hertzian initiation can be seen. Dark green smears and a globule in cracks on the backed margin, visible under low-powered magnification, is interpreted as hafting residue. Initial inspection of this pink crescent (by Richard Fullagar) suggests this tool has been used for wood-working [53]. This tool is found near the boundary of SU5 and SU6. Excavated lying flat *in situ* (B10 XU15-01) the backed artefact is within 50cm (laterally) and 4cm above the dated charcoal sample (B10 XU15-02: Wk-44287) with a median age of 43,330 cal. BP. The very early use of this specialised technology is much earlier than the modelled arid zone proliferation of adzes and microliths linked to ENSO intensification [54], and even earlier than previously identified Australian Pleistocene examples from Queensland and the Flinders Ranges [12, 55]. There is a complete absence of this distinctive raw material in the late Holocene backed artefact proliferation, and no micro-debitage in the intervening mid-Holocene and terminal Pleistocene assemblages (the closest backed artefact is in B10 XU9). This, combined with the undisturbed stratigraphy of SU6 (Fig 7), means that we believe that this early example was *in situ* (and see 54).

The Pleistocene assemblages (AU3a+b and AU4) comprise less than 10% of the total assemblage by number (but not weight). Nevertheless, persistent artefact discard through this lower part of the sequence demonstrates repeated site visits from before 47,830 cal. BP (Wk-44289; median modelled age). Four artefacts (a quartz flake frag; a chert flake frag; a retouched ironstone piece and a quartzite complete flake) plus 12 pieces of micro-debitage (mostly chert, with quartz and quartzite) were found in SU7 and SU8, below the lowest radiocarbon samples. The LGM assemblage of 370 artefacts (6 mm n = 48, 2 mm n = 322; in AU3b) associated with a date range of 25,370–17,930 cal. BP provides the first unequivocal evidence for human occupation in the Western Desert during the Last Glacial Maximum. Site visits during the terminal Pleistocene and Pleistocene/ Holocene transition phases are recorded in Square B10 (AU3a) where 1,154 artefacts are discarded (6 mm n = 208, 2 mm n = 946: 8 artefacts/kg sediment).

### Holocene lithic assemblages

Most artefacts were discarded during the Holocene (AU1), with the majority in the last millennium (78.4%, 6 mm n = 2,654, 2 mm n = 19,553). Smaller peaks and ebbs in artefact discard within this unit indicate three distinct occupation pulses. The mid-Holocene occupation (AU2) had similar artefact discard rates (per kilogram) to the most recent occupation pulses (13.8%, 6 mm n = 625, 2 mm n = 3,449), albeit spread over a much longer time period. Differences noted between the two squares in hearth and discard patterns reflect spatial variability in occupation activities across the site.

Two distinct Holocene phases also occur at Puntutjarpa [56] and provide an opportunity to compare Western Desert raw material use and technological strategies during the late Holocene.

The last millennium signals a major shift in raw material use (Fig 12). From the Pleistocene and during the mid- to late Holocene, chert comprises under half of the total assemblage (n = 1,462: 42.4%) with quartz, quartzite and silcrete also commonly used and discarded. With the marked increase in discard rates at the start of the last millennium, chert use increases significantly (χ2 (1) = 217.86, p = <0.01), while quartz, quartzite, and silcrete usage declines. This increased focus on chert during the last thousand years most likely reflects changing technological organisational practices.

All backed artefacts (bar the early specimen described above) and tula adzes were discarded during the last millennium. Proportions of other retouched/used artefacts are also highest in AU1 (n = 157: 5.9%). Backed artefact production correlates with peaks in micro debitage (Fig 13).

All grinding materials were recovered from the Holocene units and represent ‘informal’, more amorphous types typical of early to mid-Holocene Western Desert assemblages [3, 57, 58]. The earliest grindstone fragment comes from near the base of AU2 which is (R Date) modelled at 8,160 cal. BP (modelled median, base of SU4). Of the 28 cores/core fragments, all except two were found in the top two analytical units.

A number of researchers (for example, [4, 51, 59, 60–62]) have proposed that artefact diversity is inversely correlated with residential mobility. Residential sites are predicted to have hosted the widest range of subsistence activities resulting in the most diverse assemblages, effectively as mobility and transport constraints are removed. Assemblage diversity was measured in this study as the presence/absence of artefact types (e.g. backed artefacts, tula adzes, grinding material, other retouched/used tools: see Table 11) using the Shannon index (H, [63]) to account for sample size. Diversity increases through the terminal Pleistocene and Holocene into the last millennium (AU1: 0.95, AU2: 0.9, AU3a+b: 0.63, AU 4: 0.84). This suggests increasing use of this locale as a more regularly used base camp through time.

**Table 11:** Comparison of artefact types (6 mm sample).

AU	Unmodified complete flake	Debitage	Core/core fragment	Ground fragment	Tula adze/slug	Backed artefact	Other retouch/use-wear	TOTAL
**1**	787	1633	16	1	6	54	157	2654
**2**	198	380	10	2			35	625
**Pleistocene units (3a+b, 4)**	110	181	2			1	3	297
**TOTAL**	1095	2194	28	3	6	55	195	3576

The proportions of broken flake types through time can inform on variation in technological or taphonomic processes [64]. In the *Karnatukul* assemblage the proportions of broken flakes increase during the last thousand years (Table 12), including transversely broken chert flake proportions which increase significantly through the Holocene (AU1:AU2, χ2 (1) = 4.587, p = <0.05). This pattern likely reflects more intense human use of the rockshelter: transversely broken flakes are predominately caused by trampling rather than breakage during manufacture [65].

**Table 12:** Frequencies of broken chert flakes across Holocene units.

AU	Complete	Long.	Trans.	Other broken	All broken
**1**	495	118	458	368	944 (65.6%)
**2**	64	21	36	40	97 (60.2%)

### Reduction strategies

Because changes in art production indicate different signalling behaviours through time, Holocene reduction strategies were explored to investigate whether occupation was more sustained as well as more frequent during the last millennium. Here we compared metric and technological attributes on unmodified complete flakes (Table 12).

There are no marked temporal changes in flake shape, the number of dorsal scars and proportion of chert, quartzite and silcrete flakes with cortex between AU1 and AU2 (Table 13). Flat, single scar platforms dominate throughout the sequence, but an increase in platforms with more than one flake scar in AU1 is suggestive of comparatively later stage reduction. Significantly, more chert flakes have overhang removal in AU2 compared to AU1 (χ2 (1) = 11.246, p = <0.01). This indicates more careful reduction of chert during the mid-Holocene compared to the last millennium. Overall, however, metric and technological attributes indicate that the reduction intensity of all materials remained relatively constant during all Holocene occupation phases.

**Table 13:** Summary characteristics of complete chert, quartzite and silcrete flakes across Holocene units.

	Chert		Quartzite		Silcrete	
**AU**	1	2	1	2	1	2
**# of flakes**	495	64	95	41	145	72
**Mass (g)**	0.4 ± 0.4	0.4 ± 0.6	0.7 ± 1.4	0.8 ± 1.9	0.6 ± 1.1	0.7 ± 1.2
**Surface area estimate**	130 ± 101	130 ± 121.5	192 ± 198	180 ± 327	165 ± 186	172 ± 169
**Elongation index**	1.1 ± 0.7	1 ± 0.7	1.1 ± 0.6	1.1 ± 0.7	1.1 ± 0.6	1.1 ± 0.5
**Overhang removal (#, %)**	270 (42.1%) n = 641	49 (62.8%) n = 78	50 (37.9%) n = 132	20 (37%) n = 54	66 (35.7%) n = 185	22 (26.2%) n = 84
**Number of dorsal scars**	3 ± 2	3 ± 2	2 ± 1	2 ± 1	3 ± 1	2 ± 1
**Flakes with cortex (#, %)**	57 (11.5%)	4 (6.3%)	9 (9.5%)	4 (9.8%)	14 (9.7%)	1 (1.4%)
**Feather terminations (#, %)**	552 (72.2%) n = 765	67 (78.8%) n = 85	104 (71.7%) n = 145	51 (81%) n = 63	150 (75%) n = 200	75 (72.8%) n = 103

Each cell contains median and interquartile range unless otherwise stated

### Pigment

**Fig 16:**
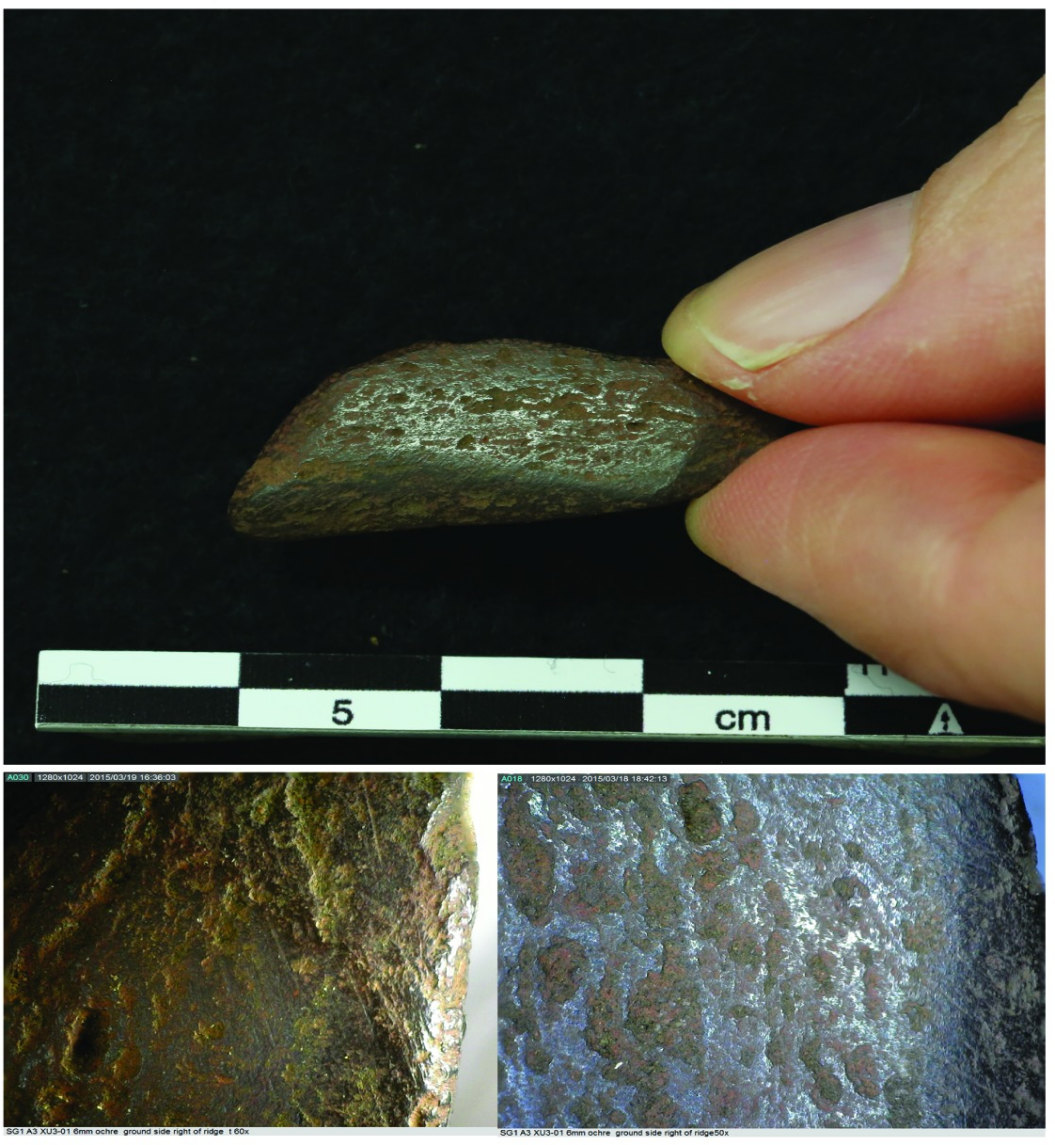
Pigment crayon from Square A3 showing microscopic wear on several facets.

Despite their location beneath major rock art panels, only minor evidence for pigment use was documented by the excavation of squares B8 and B10 (Figs 7 and 8; Table 14). One piece of dark red pigment (weighing 4.92g; from square A3 XU3) has signs of wear indicating its use either as a crayon or in the preparation of red paint (Fig 16). Less than 80g of pigment (black, cream, red, white, purple and yellow) were retrieved during the excavation and one purple ochreous block (weighing 159g) was recovered from the surface of square B10. None of the ochrous material from these two squares showed signs of use (paint preparation, paint splatter), but their presence in this deposit indicates that they were manuports.

**Table 14:** Pigment retrieved from the two analysed squares.

B8—Colour (grams)								B10—Colour (grams)						
AU	Cream	Purple	Red	White	Yellow	Total	*%f*	Black	Cream	Purple	Red	White	Total	*%f*
SUR										158.9			159	
1	3.9	39.9	1.1	2.6	0.0	47.6	*70*.*4*		0.3		0.95	5.05	6.34	*68*.*5*
2	0.0	0.8	1.2	0.2		2.1	*3*.*1*					0.2	0.2	*1*.*8*
3a						na		0.4			0.2	0.36	0.94	*10*.*2*
3b		8.4	3.4	0.0		11.8	*17*.*4*						na	
4	6.1					6.1	*9*.*0*					1.8*	1.8	*19*.*5*
Total	10.0	49.0	5.7	2.8	0.0	67.6		0.4	0.3		1.1	7.4	9.3	*100*

*these nodules could be gypsum–like by products from the highly cemented/altered lower strata

Around 70% of the excavated pigment–and the most variability in colour use–was found in the deposits dated to the last millennium (AU1). Very small amounts of pigment (c. 2g) were retrieved from the units dated to the mid Holocene (AU2): and these were red, purple and white. Intriguingly both the terminal Pleistocene and glacial units (AU3a and AU3b) retuned around five times the quantities of pigment found in the mid-Holocene units.

## The contemporaneity of occupation and art

The stone artefacts discarded at *Karnatukul* represent a long-term record of artefact use, transport and discard. Chert and chalcedony were more intensively reduced and conserved compared to other raw material classes. Low frequencies of cortex on debitage and on cores, in all material classes, indicate that nodules were consistently carried into this site at a later stage of reduction. Some larger cortical flakes and cores may have been taken away as people left the site in anticipation of future use. This pattern suggests high residential mobility: an important strategy in the human use of arid environments. Frequent group movements around the Western Desert were necessary to schedule critical resource use, but this placed limitations on what could be transported. In these situations, the use and conservation of high quality, predictable flaking stone and an emphasis on small portable toolkits with reliable, maintainable and multi-functional tools (such as backed artefacts) likely alleviated mobility constraints [66–68]. Changes in lithic technology and site use at *Karnatukul* reflect wider and regional changes in mobility and settlement patterns through time.

The *Karnatukul* pre-glacial Pleistocene assemblage (AU4) has evidence for a range of materials being reduced on-site with an early preference for high quality chert. All materials are likely available within the wider upland catchment, although not in immediate proximity to the site. This assemblage includes a retouched ironstone scraper and a backed artefact. This backed artefact, in deposits dated to 45,570–41,650 cal. BP; Wk-44287) with evidence of a break potentially suffered in a haft, signifies very early experimentation in this specialised technology in Australia by highly mobile foragers. The presence of an even earlier snapped scraper, which also appears to have suffered a break while hafted, provides additional evidence that very early arid zone peoples were practising specialised hafting technologies. No cores were recorded from this early occupation phase, which is likely a sample size issue.

The only surviving rock art in the *Karnatukul* shelter likely to have been produced during the earliest occupation by Pleistocene peoples [69] includes heavily weathered cupules and abraded grooves, located low on a sloping back wall, just above the current floor level. *Katjarra* has a small number of heavily weathered engraved track and geometric motifs which likely represent earlier and more mobile Pleistocene art producers [19]. Currently undated, these will be sampled for U-Th crusts over the next several years. Like the earliest evidence for occupation documented here, this assumed early art production was likely episodic and appears to represent the earliest symbolic mapping by the first people venturing into this landscape: signalling open social networks.

Artefact discard rates increase slightly during the glacial and terminal Pleistocene period (AU3a and b) and indicate continued–albeit episodic–site visits by highly residentially-mobile groups. This is significant, as dates for the LGM have been absent from the Western Desert until this excavation. Few cores and tools were discarded during this time, again, likely a sampling issue.

Two distinct pulses of artefact discard occur during the Holocene at *Karnatukul*. The first, which is marked by an increase in assemblage diversity, occurs in the mid Holocene (AU2). Cores and retouched/used artefacts become more common and grinding technologies appear in the sequence for the first time. The presence of grindstone base fragments demonstrates increased variety in activities, such as animal, plant or seed processing [70–72]. The toolkit carried by people visiting *Karnatukul* during the mid-Holocene appears to be designed for a more generalised rather than a specialised subsistence economy (cf. [73]). Tools were made on a wide range of flake blank sizes and shapes which were multifunctional and flexible for different tasks.

Occupation during the last millennium at *Karnatukul* is marked by a distinct change in the archaeological record, likely reflecting a regional shift in settlement patterns. Artefact discard rates increase substantially and the use of chert as the favoured material for tool manufacture increases significantly over silcrete, quartzite and quartz. The increase in 6 mm discard rates is matched by peaks in the 2 mm artefact fraction. Chert micro-debitage dominates the assemblage, signalling the technological change to specialised tula and backed artefact production, demonstrating that on-site manufacture, maintenance and discard of backed artefacts took place at *Karnatukul*. Shott [60] and Veth [4] suggest that as settlement mobility decreases, an increase in material-culture inventories may reflect non-utilitarian contexts related to the transfer of messages about social affiliation and status. The exotic tektite and proliferation of backed artefacts discarded at *Karnatukul* during this period may have been linked to social signalling as well as functional factors (also see [74]).

Many aspects of the *Karnatukul* assemblage resonate with broader regional patterns found across inland arid Australia during the late Holocene. Increased intensity of site occupation, a preference for high quality stone and increasing conservation strategies are key trends seen at other Western Desert [57, 58, 75] and indeed central Australian sites [9, 76, 77]. However, Western Desert sites such as Bush Turkey 3 [10] and Kaalpi [58] show increased residential mobility and logistical provisioning during the last millennia with visits to these places becoming more frequent but of shorter duration. At *Karnatukul*, in contrast, there is a proliferation event in this time frame. The *Karnatukul* assemblage is unique because of the sheer quantity of debris and the high frequencies of tool discard–particularly of formal, long-use life tools such as tula adzes and backed artefacts. A similar technological trend is observed at Puntutjarpa, with all the tulas and the single backed artefact occurring in Zone A. But at Puntutjarpa this has been described as “a veneer of last millennial material” ([57]: Fig 6). Western Desert sites demonstrate considerable variability in individual site histories, speaking to the far greater complexity in the use of the arid landscape than was previously recognised ([2] and see; [78]).

There are no significant changes in raw material selection at *Karnatukul* after the LGM although chert and chalcedony become more intensively reduced than other materials, particularly in the Holocene. This pattern accords well with the recent observations made at Puntutjarpa ([57] pp. 29). But again, this pattern is different to that found at Bush Turkey 3, where there is a marked decrease in the proportion of artefacts with cortex and in artefact size in the recent past [10]. The *Karnatukul* assemblage suggests not only a marked increase in frequency in use of this locale, but a transition from a satellite site visited episodically, perhaps as an aggregation locale for the larger group, to a more regularly visited and territorially tethered ‘home-base’ where intensive reduction and tool manufacture took place.

**Fig 17:**
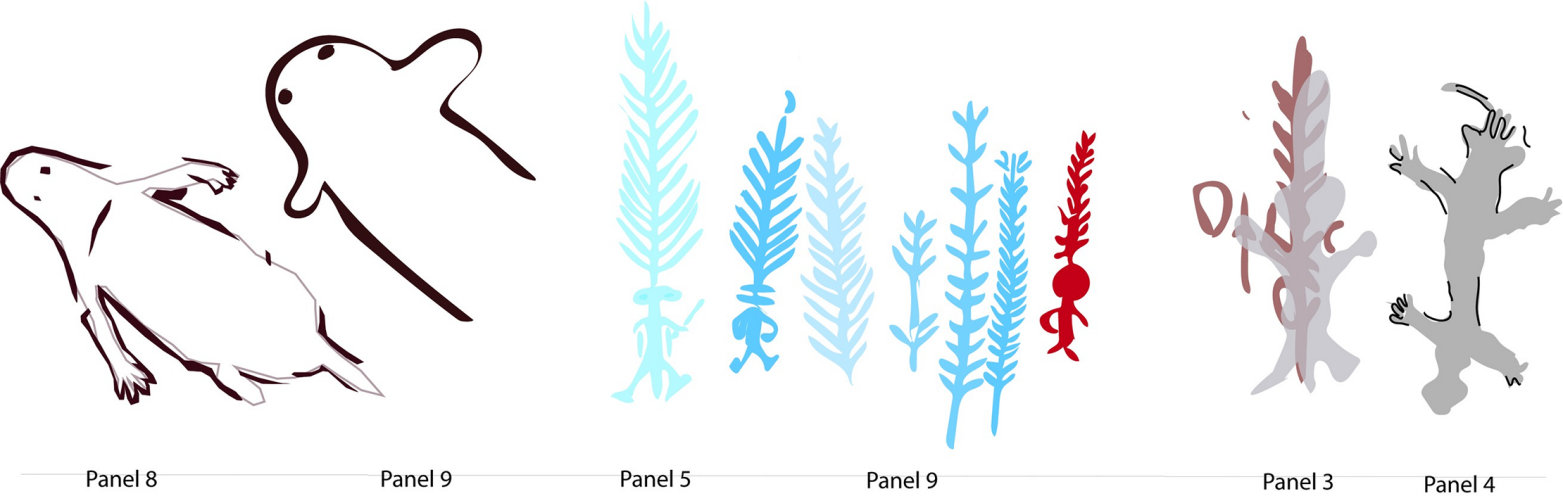
*Karnatukul* human figures: (left) Phase 1 bichrome; (centre) white and red headdress figures; (right) Phase 5 monochrome figures dated to the last millennium, over Phase 4 red geometrics (Panel 3) and with subsequent black drawn outline (Panel 4).

**Fig 18:**
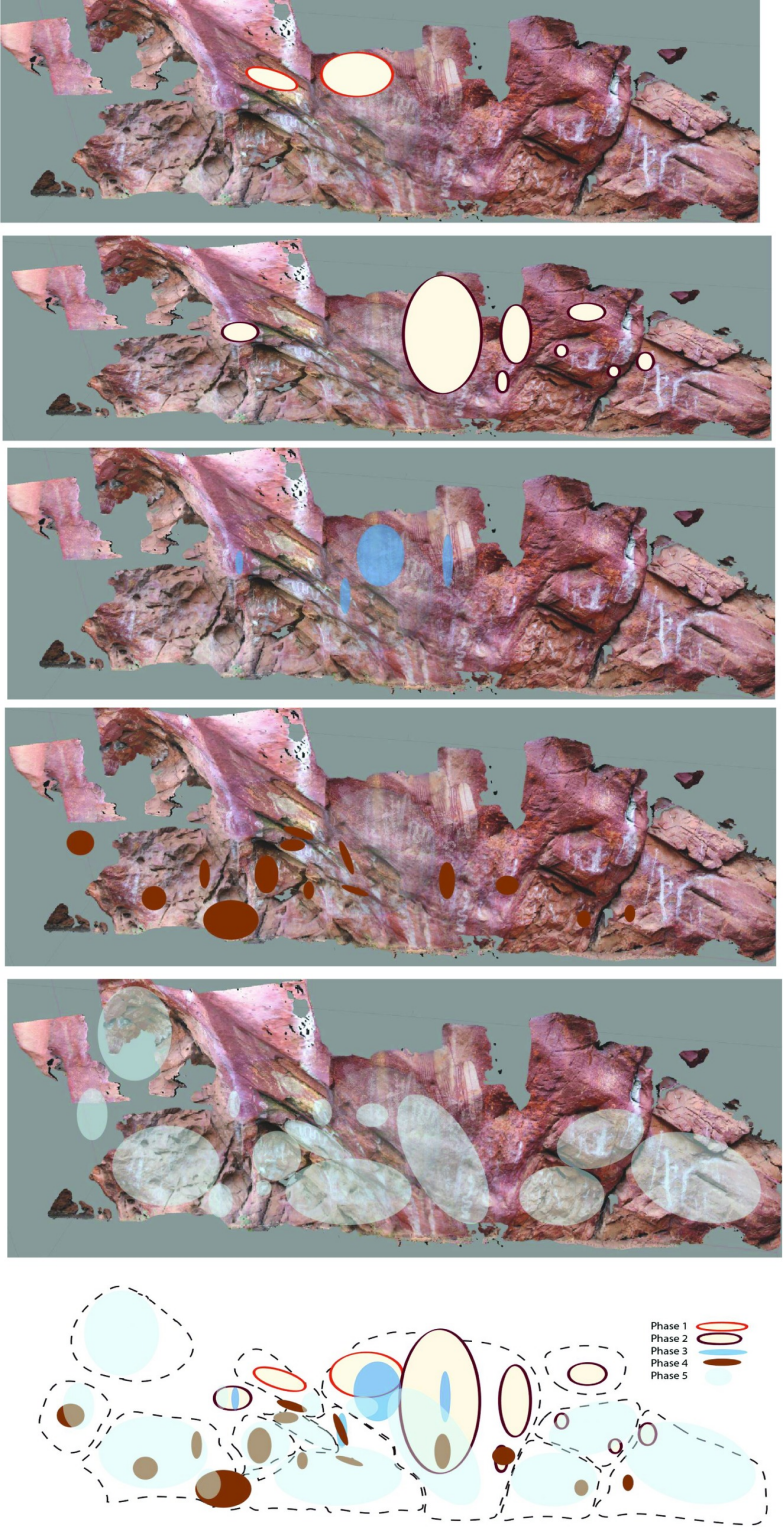
Differential placement of phased art production within the *Karnatukul* site.

The art production at the site changes in nature and placement over time. Both the stylistic discontinuities between the art phases (Fig 17), and the changed placement of these within the site (Fig 18), support these phases as being temporally discrete. Only the most art production (Phase 5) has been dated (see above).

The earliest pigment art is judged as being contemporaneous with the mid-Holocene phase of occupation, modelled now between 8,160–2,330 cal. BP. This first art production was emblemic: with a highly-visible complex composition including distinctive human figures and large geometric motifs which may be ceremonial objects or decorated shields. The location of this composition high on the wall indicates art production with narrative signalling intent. No other composition like this has been recorded in the Western Desert, although motifs from this same style: i.e. large bichrome red and white human figure and decorated oval shapes–are also found in the nearby mid-Holocene *Wirrili* site, where the main occupation focus occurred between 4,500–5,500 cal. BP [54]. During this time, the shelter was occupied by people undertaking a generalised range of stone tool activities which were multifunctional and flexible. The artists who produced pigment art at this time signalled to assert their identity.

This initial pigment phase is replaced by a subsequent pigment production which appears to represent corporate identity for the broader *Katjarra* region: headdress figures. This signalling vocabulary is found widely across the Western Desert [79]. Headdress figures: diminutive linear humans with large distinctive fern-shaped headdress are found around the Western Desert, but the *Katjarra* style (with an elongated symmetrical fern design) is peculiar to this specific rangeland and distinguishable from the headdress figures produced by other territorially tethered groups [19]. Headdress figures have proved elusive to direct-date because of the use of red and white pigment in their making. They are modelled to have proliferated during Phase 6 of the Western Desert art chronology [70]. This phase of art production is superimposed directly over the earlier composition and other panels suggesting symbolic replacement, not coincidence [80]. The absence of headdress designs recorded at other Western Desert Ranges (i.e. *Jilakurru*, *Kaalpi* and *Mungulu* [19]) suggests that *Katjarra* is the homeland of a territorially circumscribed group moving between desert upland and flanking lowlands, asserting that group identity through this motif form. The stylistic variability found in the *Karnatukul* headdress figures represents the full range of variability found around *Katjarra*, suggesting that *Karnatukul* served as an aggregation locale for this group during this time period [81, 82]. This mid-late Holocene art production event cannot be easily correlated with the occupation evidence since there is minimal occupation evidence for this time period. Indeed, the shelter may have had a different type of occupation focus at this time, with domestic activities perhaps located on the sand plain outside the rockshelter locale. This pattern of alternating site use would result in pulses of contemporaneous art and occupation evidence followed by intervening periods when art and/or occupation was not produced as dual actions within the rockshelter.

Group-identity signalling is replaced during the most recent phases of art production by art which signals local-group identity. The spatial distribution (Fig 18) and the demonstrable (dated) association with the intensive occupation as a living place, reflects a domestic focus in the shelter’s use during the most recent art production. It has been argued elsewhere [83] that the art that people produce in their living sites is produced for an audience which includes the entire social group encountered during their daily economic activities. This is different to art created in a more restricted context, i.e. where the audience is limited to particular individuals/genders or social groups, e.g. for ceremonial purposes. The focus on this place for habitation, demonstrated by a conspicuous increase in its use for artefact manufacture and discard (and richness indices) demonstrates lower residential mobility. In such a location social mechanisms are needed to mediate possible conflict. Increased sedentism and social proscription can result in a proliferation of symbolic behaviours, and particularly those which demonstrate local group social affiliation [84]. The rock art production during the last millennium supports this picture of a localised home-base and its spatial placement across the shelter demonstrates art production as one of a range of secular activities.

These changing symbolic practices occurred within an emerging late Holocene system of aggregation and social coalescence within the Carnarvon Ranges. *Karnatukul* is a classic small desert refugium [4, 9] which demonstrates a changing focus of human symbolic practice through its deep time occupation.

## Conclusions

*Karnatukul* provides evidence for the earliest occupation of the Australian Western Desert, at a modelled age of 50,010 cal. BP– 45,190 cal. BP (median age 47,830 cal. BP), pushing back the previous known occupation of this Western Desert site by more than 20,000 years. This dated sequence is clearly at the edge of the radiocarbon barrier, with the site’s heavily cemented basal aggregate units resistant to OSL sampling. The C_14_ dates are consistent with early dates from the wider arid zone including Boodie Cave on the North West Shelf [84, 85], Riwi and Parnkupirti at the Kimberley edge/Great Sandy Desert interface [53, 11] as well as at Waratayi, on the desert’s edge, to the east in the northern Flinders Ranges [12]. Collectively these sites provide OSL and radiocarbon ranges which statistically overlap with the date range (c. 49,000 to 45,000 cal. BP): that range conservatively mooted [86] for the first occupation of Sahul. These sites make a persuasive case for the onset of Aboriginal occupation well before this threshold age [7, 87]. These sites document the earliest human occupation of the Australian arid zone from contexts as varied as the maritime desert coastline, the savannah desert interface, southern range uplands and now rangelands from amongst interior linear sand dunes.

*Karnatukul* provides the earliest evidence for human occupation of Australia’s desert core offering increased understanding of early arid zone human behaviours. The site registers low levels of episodic occupation throughout the Pleistocene. A single geometric backed artefact provides the earliest evidence in Australia for experimentation with this specialised technology. The revised *Karnatukul* sequence provides the first unequivocal evidence for persistence of occupation of the Western Desert during the Last Glacial Maximum [contra 9, 57], as well as an occupation pulse during the late Pleistocene/early Holocene transition. There are two different-scale proliferation events identified in the Holocene: first during the mid-Holocene, then with the most intensive occupation during the last millennium. The sequence recalibrates important features of the arid zone time-series data [5] and confirms aspects of the relationships between site occupational intensity and phases of climatic amelioration. Superimposition and style analyses using Harris matrices and spatial placement demonstrate a robust sequence of stylistic change in pigment art production at this site. While we have not yet been able to direct-date the oldest pigment style phases, we have AMS direct-dated the most recent pigment assemblage.

The excavated sequence and direct-dating of the pigment art confirms the contemporaneity of multiple social actions during the last millennium. *Karnatukul* was a regularly visited ‘home-base’ where people both intensively made and used stone tools, as well as creating pigment art which signalled local-group identity. The combined art and occupation sequences suggest that as well as the occupation focus of this place having changed through time, that the types of signalling behaviours being deployed during the Holocene also changed. Correlating symbolic behaviour with other occupation evidence in the recent past demonstrates the changing dynamics in arid zone behaviours through time.

*Karnatukul* provides significant new evidence for the early and successful adaptation of modern peoples to the interior deserts of Australia. Importantly, this site adds to other arid and semi-arid zone sites recently dated to between 49,000–45,000 cal. BP across the northwest and central-south of the continent. These results push back this time frame to the edge of the radiocarbon barrier and necessarily confirm the minimum age for the occupation of Sahul [88]. This classic desert site demonstrates a changing focus of human symbolic practice in the recent past, and provides a fuller comprehension of the complexity and dynamism of desert peoples through deep time. Important technological innovation registered in this assemblage highlights the dynamic adaptive culture of the first Australians, and supports arguments for their rapid movement from the northern tropics both around the coast and through the arid interior of the continent.

## References

[1] O'Connor S, Veth PM and Campbell C. Serpent's Glen: a Pleistocene archaeological sequence from the Western Desert. Australian Archaeology. 1998; 46: 12–22.

[2] Gould RA. Puntutjarpa Rockshelter and the Australian desert culture. Anthropological Papers American Museum of Natural History. 1977; 54: 1–187.

[3] Smith MA. The pattern and timing of prehistoric settlement in Central Australia, PhD. Thesis, University of New England. 1988.

[4] Veth PM. Islands in the Interior: The Dynamics of Prehistoric Adaptations within the Arid Zone of Australia. Michigan: Ann Arbor Press; 1993.

[5] Williams AN, Veth PM, Steffen W, Ulm S, Turney CSM, Reeves JM, et al A continental narrative: Human settlement patterns and Australian climate change over the last 35,000 years. Quaternary Science Reviews. 2015; 123: 91–112.

[6] McDonald JJ, Steelman KL, Veth PM, Mackey J, Loewen J, Thurber CR, Guilderson TP. Results from the first intensive dating program for pigment art in the Australian arid zone: insights into recent social complexity. Journal of Archaeological Science. 2014; 46: 195–204.

[7] Clarkson C, Jacobs Z, Marwick B, Fullagar R, Wallis L, Smith M et al Human Occupation of northern Australia by 65,000 years ago. Nature. 2017; 547: 306–310 20 July 2017. 10.1038/nature22968 28726833

[8] Veth PM, Ward I, Manne T, Ulm S, Ditchfield K, Dortch J et al Early human occupation of a maritime desert, Barrow Island, North-West Australia. Quaternary Science Reviews. 2017; 168: 19–29.

[9] Smith MA. The Archaeology of Australia’s Deserts. Cambridge: Cambridge University Press; 2013.

[10] Veth PM, McDonald JJ & White E. Dating of Bush Turkey Rockshelter 3 in the Calvert Ranges establishes Early Holocene Occupation of the Little Sandy Desert, Western Australia. Australian Archaeology. 2008; 66: 33–44.

[11] Veth PM, Smith MA, Bowler J, Fitzsimmons KE, Williams AN and Hiscock P. Excavations at Parnkupirti, Lake Gregory, Great Sandy Desert: OSL ages for occupation before the Last Glacial Maximum. Australian Archaeology. 2009; 69: 1–10.

[12] Hamm G, Mitchell P, Arnold LJ, Prideaux GJ, Questiaux D, Spooner NA, et al Cultural innovation and megafauna interaction in the early settlement of arid Australia. Nature. 2016; vol. 539, no. 7628: 280–283. 10.1038/nature20125 27806378

[13] Law WB, Cropper DN & Petchey F. Djadjiling Rockshelter: 35,000 14C years of Aboriginal Occupation in the Pilbara, Western Australia. Archaeology in Oceania. 2010; 70: 68–71.

[14] Morse K, Cameron R. & Reynen W. A tale of three caves: New dates for Pleistocene occupation in the inland Pilbara. Australian Archaeology. 2014; 79: 167–178.

[15] Slack M, Fillios M & Fullagar R. Aboriginal Settlement during the LGM at Brockman, Pilbara Region, Western Australia. Archaeology in Oceania. 2009; 44: 32–39.

[16] Tonkinson R. The Jigalong Mob: Aboriginal victors of the Desert Crusade. California: Cummings Publishing Co; 1974.

[17] Veth PM. Statement of Archaeological Evidence in the Martu Native Title Claim. Report Prepared for the Ngaanyatjarra Aboriginal Corporation. Alice Springs. 2001.

[18] McDonald JJ and Veth PM. Birriliburu IPA: A Report on the Archaeological Values. Report to Central Desert Native Title Services on behalf of Mungarlu Ngurrarankatja Rirraunkaja, UWA, Perth. 2012a.

[19] McDonald JJ. Discontinuities in arid zone rock art: Graphic indicators for changing social complexity across space and through time. Journal of Anthropological Archaeology. 2017; 46: 53–67 10.1016/j.jaa.2016.08.005

[20] Harris E and Gunn RG. The use of Harris Matrices in rock art research. Oxford Handbook of Anthropology and Archaeology of Rock Art. 2016; Oxford Handbooks Online.

[21] Green H, Gleadow A, Finch D, Hergt J and Ouzman S. Mineral Deposition Systems with Rock Art Sites, Kimberley, NW Australia—Field Observations. Journal of Archaeological Science Reports. 2017; 14: 340–352.

[22] Steelman KL, Rowe MW. Radiocarbon Dating of Rock Paintings: Incorporating pictographs into the archaeological record In: McDonald JJ, and Veth PM, editors. A Companion to Rock Art, Blackwell CoRmpanions to Anthropology series. Oxford: Wiley-Blackwell Publishing, 2012 pp.565–582.

[23] Harman J. Image Enhancement using DStretch http://www.dstretch.com/DStretchHandout.pdf (accessed 12 January 2018).

[24] Wentworth CK A Scale of Grade and Class Terms for Clastic Sediments. The Journal of Geology. 1922; 30 (5): 377–392.

[25] Rayment GE and Lyons DJ Soil Chemical Methods—Australasia Australian Soil and Land Survey Handbooks Series. Collingwood: CSIRO Publishing 2010.

[26] Goldberg P and Macphail RI Practical and Theoretical geoarchaeology. Victoria, Blackwell Publishing Ltd 2006. Print ISBN:9780632060443

[27] Vandenberghe J Grain size of fine-grained windblown sediment: A powerful proxy for process identification. Earth-Science Reviews. 2013; 121:18–30.

[28] Tite MS and Mullins C Enhancement of the magnetic susceptibility of soils on archaeological sites. Archaeometry. 1971; 13(2):209–219.

[29] Asouti E and Austin P. Reconstructing woodland vegetation and its exploitation by past societies, based on the analysis, interpretation of archaeological wood charcoal macro-remains. Environnemental Archaeology. 2005; 10: 1–18.

[30] Dotte-Sarout E, Carah X, and Byrne C. Not just carbon: assessment and prospects for the application of anthracology in Oceania. Archaeology in Oceania. 2015; 50 (1): 1–22. 10.1002/arco.5041

[31] Thery-Parisot I, Chabal L and Chravzez J. Anthracology and Taphonomy: from wood gathering to charcoal analysis. A review of the taphonomic processes modifying charcoal assemblages, in archaeological contexts. Palaeogeography, Palaeoclimatology, Palaeoecology. 2010; 291: 142–153.

[32] Chabal L, Fabre L, Terral JF and Théry-Parisot I. L’anthracologie. L'étude paléoécologique de sites protohistoriques à partir des charbons de bois: la question de l'unité de mesure-Dénombrements de fragments ou pesées? In: Bourquin-Mignot C, Brochier JE, Chabal L, Crozat S., Fabre L., et al editors. Paris: La Botanique; 1999, pp.43–104.

[33] Latz P. Bushfires and Bushtucker: Aboriginal plant use in Central Australia. Alice Springs: IAD Press 1995.

[34] Low T. Wild Food Plants of Australia. Sydney: Harper Collins Publishers; 1991.

[35] Whitau R, Vannieuwenhuyse D, Dotte-Sarout E, Balme J, O'Connor S Home is where the hearth is: anthracological and microstratigraphic analyses of Pleistocene and Holocene combustion features, Riwi Cave (Kimberley, Western Australia). Journal of Archaeological Method and Theory. 2017 10.1007/s10816-017-9354-y PMC6061027 30100699

[36] Bronk Ramsey C. Bayesian analysis of radiocarbon dates. Radiocarbon. 2009a; 51: 337–360.

[37] Hogg AG, Hua Q, Blackwell PG, Niu M, Buck CE, Guilderson TP, et al, SHCal13 southern hemisphere calibration, 0–50,000 years cal BP. Radiocarbon. 2013; 55: 1889–1903.

[38] Waikato Radiocarbon AMS Technical Report 2017.

[39] Bronk Ramsey C. Deposition models for chronological records. Quaternary Science Reviews. 2008; 27: 42–60.

[40] Bronk Ramsey C. Dealing with outliers and offsets in radiocarbon dating. Radiocarbon. 2009b; 51: 1023–1045.

[41] Akerman K. The use of australites for the production of implements in the western desert of Western Australia. Occasional Papers in Anthropology, University of Queensland 1975; 4: 117–123.

[42] McNamara K and Bevan A. Tektites (3rd edition). Western Australia: Western Australian Museum; 2001.

[43] Rowland B. An investigation of the use of Australites (tektites) at Olympic Dam, South Australia. Journal of the Anthropological Society of South Australia. 2014; 38: 136–154.

[44] Baker G. The role of australites in Aboriginal customs. Memoirs of the National Museum of Victoria. 1957; 22:1–23.

[45] Berndt RM and Berndt CH. A preliminary report of fieldwork in the Ooldea region, western South Australia. Oceania. 1943/44; 14(4): 51–57.

[46] Bevan AWR and Bindon P. Australian Aborigines and meteorites. Records of the Western Australian Museum 1996; 18: 93–101.

[47] de Lombera-Hermida A., Rodríguez-Rellán C. Quartzes matter. Understanding the technological and behavioural complexity in quartz lithic assemblages. Quaternary International. 2016; 424: 2–11.

[48] Hiscock P. Mobility and technology in the Kakadu coastal wetlands. Bulletin of the Indo-Pacific Prehistory Association. 1996; 15: 151–157.

[49] Veth PM, Hiscock P and Williams AN. Are Tulas and ENSO linked in Australia? Australian Archaeology. 2011; 72: 7–13.

[50] Andrefsky W. Lithics: macroscopic approaches to analysis. 2nd ed. Cambridge, New York: Cambridge University Press 2005.

[51] Clarkson C. Lithics in the land of the lightning brothers the archaeology of Wardaman Country, Northern Territory Terra Australis 26 Canberra: ANU EPress, 2007.

[52] Wood R, Jacobs Z, Vannieuwenhuyse D, Balme J, O’Connor S and Whitau R. Towards an accurate and precise chronology for the colonization of Australia: The example of Riwi, Kimberley, Western Australia. PLoSONE. 2016; 1–25. 10.1371/journal.pone.0160123 27655174 PMC5031455

[53] McDonald J, Reynen, Fullagar R. Testing predictions for symmetry, variability and chronology of backed artefact production in Australia’s Western Desert. Archaeology in Oceania. 2018; 1–12. 10.1002/arco.5162

[54] Hiscock P. Pattern and context in the Holocene proliferation of backed artefacts in Australia In: Elston RG Kuhn S (eds) Thinking Small: Global perspectives on microlithization Archaeological Papers of the American Anthropological Association Number 12, pp. 163–178. 2002 American Anthropological Association, Indiana.

[55] Slack MJ, Fullagar RL, Field JH & Border A. New Pleistocene Ages for Backed Artefact Technology in Australia. Archaeology in Oceania. 2004; 39: 131–137.

[56] Smith MA, Williams AN and Ross J. Puntutjarpa rockshelter revisited: a chronological and stratigraphic reappraisal of a key archaeological sequence for the Western Desert, Australia. Australian Archaeology. 2017; 83 (1–2): 20–31.

[57] Veth PM, Smith MA, Haley M. Kaalpi: The archaeology of a sandstone outlier in the Western Desert. Australian Archaeology. 2001; 52: 9–17.

[58] Veth PM. Cycles of aridity and human mobility: risk minimisation among late Pleistocene foragers of the Western Desert, Australia In: Veth PM, Smith M and Hiscock P, editors. Desert Peoples: Archaeological Perspectives. Oxford: Blackwell Publishing; 2005 pp. 100–115.

[59] Shott M. Technological organisation and settlement mobility: an ethnographic examination. Journal of Anthropological Research 1986; 42(1): 15–51.

[60] Kuhn SL and Clark AE. Artifact densities and assemblage formation: Evidence from Tabun Cave. Journal of Anthropological Archaeology. 2015; 38: 8–16.

[61] Nelson MC. The Study of Technological Organization. Archaeological Method and Theory. 1991; 3: 57–100.

[62] Riel-Salvatore J and Barton CM. Late Pleistocene Technology, Economic Behaviour and Land-Use Dynamics in Southern Italy. American Antiquity. 2004; 69(2): 257–274.

[63] Hammer Ø. PAST: Paleontological Statistics Version 3.0 Reference Manual. Oslo: Natural History Museum, University of Oslo; 2013.

[64] Hiscock P. Quantifying the Size of Artefact Assemblages. Journal of Archaeological Science. 2002; 29(3): 251–258.

[65] Hiscock P. The need for a taphonomic perspective in stone artefact analysis. Queensland Archaeological Research. 1985; 2: 82–95.

[66] Andrefsky W. Human Land-use Strategies and Projectile Point Damage, Resharpening, and Discard Patterns. Human Evolution. 2010; 25: 13–30.

[67] Kelly RL. The Three Sides of a Biface. American Antiquity. 1988; 53(4): 717–734.

[68] Kuhn SL. A formal approach to the design and assembly of mobile toolkits. American Antiquity. 1994; 59 (3): 426–442.

[69] McDonald JJ and Veth PM. Rock art in arid landscapes: Pilbara and Western Desert petroglyphs. Australian Archaeology. 2013; 77: 66–81.

[70] Fullagar R, Stephenson B, Hayes E. Grinding grounds: Function and distribution of grinding stones from an open site in the Pilbara, Western Australia. Quaternary International. 2017; 427: 175–183.

[71] Hayes E, Fullagar R, Mulvaney K and Connell K. Food or fibercraft? Grinding stones and Aboriginal use of Triodia grass (spinifex). Quaternary International. 2018; 468B: 271–283 10.1016/j.quaint.2016.08.010

[72] Reynen W and Morse K. Don’t forget the fish–towards an archaeology of the Abydos Plain, Pilbara, Western Australia. Australian Archaeology. 2016; 82: 94–105.

[73] Loyola R, Cartajena I, Núñez L. & Patricio López M. Moving into an arid landscape: Lithic technologies of the Pleistocene–Holocene transition in the high-altitude basins of Imilac and Punta Negra, Atacama Desert. Quaternary International. 2017; DOI.10.1016/j.quaint.2017.10.010 10.1016/j.quaint.2016.09.045

[74] Hiscock P. Horizons of change: entanglement of palaeoenvironment and cultural dynamic in Australian lithic technology In: Robinson E and Sellet F editors. Lithic Technological Organisation and Paleoenvironmental Change. New York: Springer 2017. pp.

[75] Veth PM. Social Dynamism in the Archaeology of the Western Desert In: David B, McNiven IJ and Barker B. editors. The Social Archaeology of Indigenous Societies. Canberra: Australian Aboriginal Studies Press 2006, pp. 242–253.

[76] Thorley PB. Shifting location, shifting scale: a regional landscape approach to the prehistoric archaeology of the Palmer River catchment, Central Australia. Unpublished PhD Thesis, Northern Territory University. 1998.

[77] Smith MA and Ross J. What happened at 1000–1500 BP in Central Australia: timing, impact and archaeological signatures. Holocene. 2008; 18(3): 387–396.

[78] Marwick B, Hiscock P, Sullivan M, Hughes P. Landform boundary effects on Holocene forager landscape use in arid South Australia. Journal of Archaeological Science Reports. 2018; 19: 864–874. 10.1016/j.jasrep.2017.07.004

[79] McDonald JJ. Archaic faces to headdresses: the changing role of rock art across the arid zone In:Veth PM Smith M and Hiscock P. editors. Desert Peoples: Archaeological Perspectives. Oxford, Blackwell Publishing Ltd 2005, pp. 116–141.

[80] Kaiser DA & Keyser JD. Symbolic superimposition: overlapping Shield Bearing Warriors at Bear Gulch. American Indian Rock Art. 2008; 34: 37–59.

[81] Conkey MW. The Identification of Hunter-Gatherer Aggregation sites—the case of Altamira. Current Anthropology. 1980; 21(5): 609–630.

[82] McDonald JJ and Veth PM. The social dynamics of aggregation and dispersal in the Western Desert In: McDonald JJ and Veth PM, editors. A Companion to Rock Art: Blackwell Companions to Anthropology Series. Oxford: Blackwell Publishing 2012,. pp. 90–102.

[83] McDonald JJ. Dreamtime Superhighway: An analysis of Sydney Basin rock art and prehistoric information exchange Terra Australis 27 Canberra: ANU EPress, 2008. ISBN 9781921536168 (pbk) 9781921536175 (pdf).

[84] Veth PM, Ditchfield K and Hook F. Maritime deserts of the Australian northwest. Australian Archaeology. 2014; 79:156–166.

[85] Ward I, Veth P, Prossor L, Denham T, Ditchfield K, Mann T. et al 50,000 years of archaeological site stratigraphy and micromorphology in Boodie Cave, Barrow Island, Western Australia. Journal of Archaeological Science Reports. 2017; 15: 344–369.

[86] Allen J, O'Connell JF. Half-right: updating the evidence for first human arrivals in Sahul. Australian Archaeology. 2014; 79: 86–108.

[87] Veth PM. Breaking through the radiocarbon barrier: Madjedbebe and the new chronology for Aboriginal occupation of Australia. Australian Archaeology. 2017; 83(3): 165–167. 10.1080/03122417.2017.1408543

[88] Norman K, Inglis J, Clarkson C, Tyler Faith J, Shulmeister J. and Harris D. An early colonisation pathway into northwest Australia 70–60,000 years ago. Quaternary Science Reviews. 2017; 180: 229–239.

