# Correction: *Karnatukul* (Serpent's Glen): A new chronology for the oldest site in Australia's Western Desert

**DOI:** 10.1371/journal.pone.0205617

**Published:** 2018-10-02

**Authors:** The PLOS ONE Staff

Figs 3, 11 and 14 are incorrect. Please view the correct figures here. The publisher apologizes for this error.

**Fig 3:**
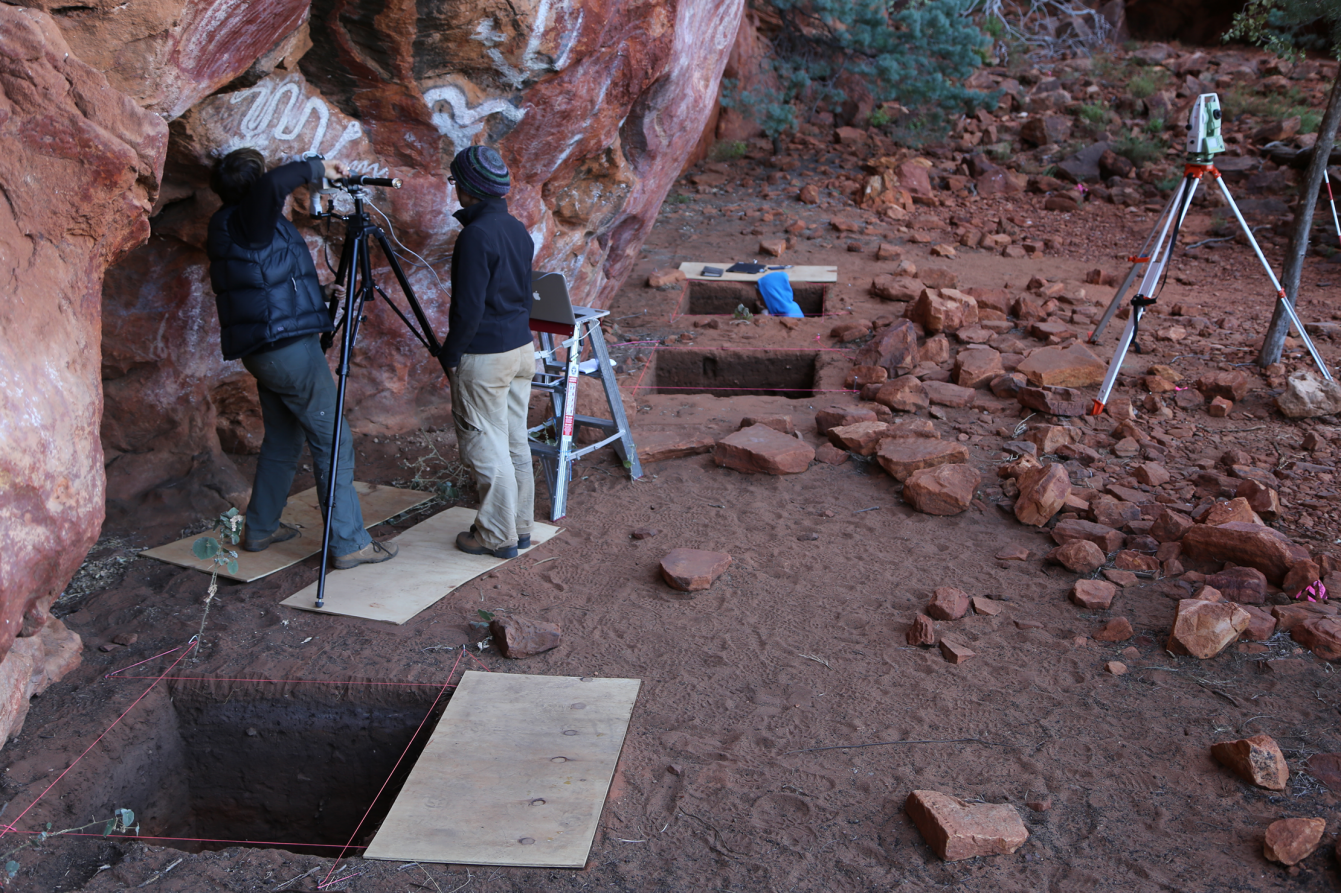
*Karnatukul* showing the nature of the site’s occupation floor and its surrounds during excavation (Photograph Peter Veth).

**Fig 11:**
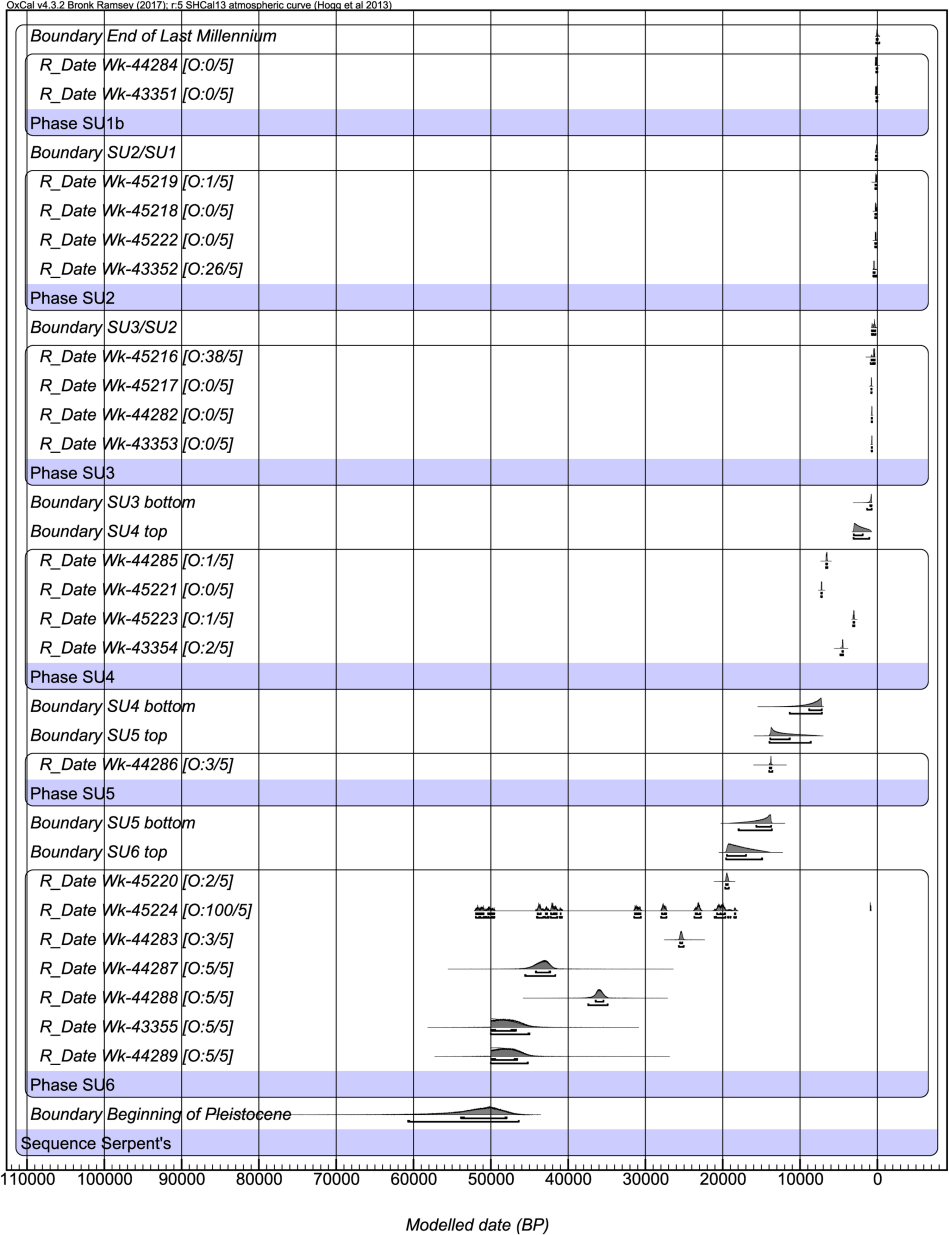
Bayesian sequence model results for the four major occupational phases at *Karnatukul*. 68% and 95% error margins are indicated by bars under each posterior age distribution.

**Fig 14:**
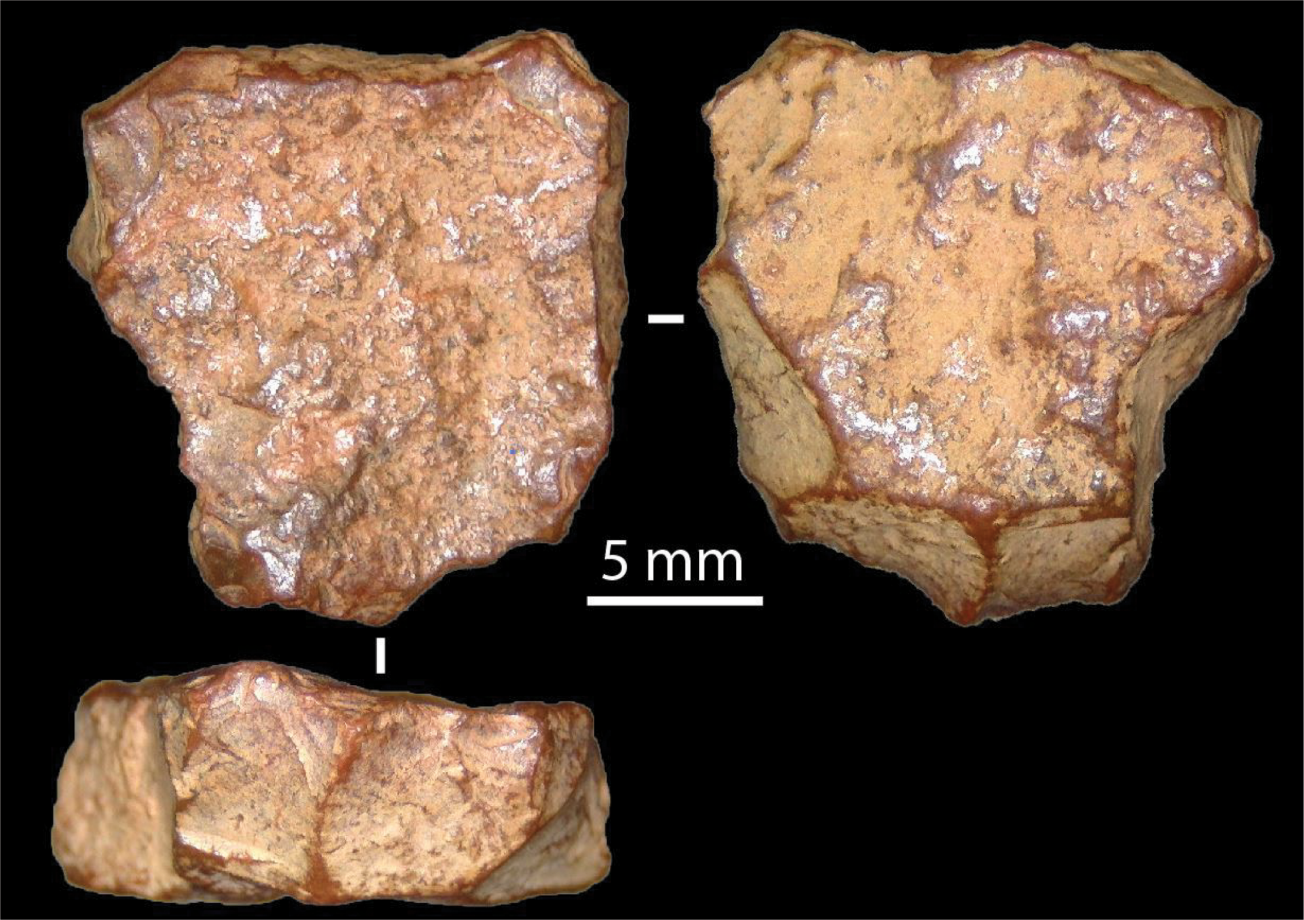
Views of l) ventral and (r) dorsal surfaces, chemically altered, of ironstone scraper (artefact B0822002).

## References

[1] McDonald J, Reynen W, Petchey F, Ditchfield K, Byrne C, Vannieuwenhuyse D, et al (2018) *Karnatukul* (Serpent’s Glen): A new chronology for the oldest site in Australia’s Western Desert. PLoS ONE 13(9): e0202511 10.1371/journal.pone.0202511 30231025 PMC6145509

